# Stage-Dependent Metabolic Responses to Oral Nutritional Supplementation in Cancer Cachexia: A Single-Arm Pilot Study

**DOI:** 10.3390/nu18040597

**Published:** 2026-02-11

**Authors:** Sora Kang, Harin Sim, Samantha O’Keeffe, Junyoung O. Park, Wonhyoung Seo, Jeong Suk Koh, Myung-Won Lee, Ik-Chan Song, Hyo-Jin Lee, Deog-Yeon Jo, Yea Eun Kang, Hyon-Seung Yi, Hyewon Ryu

**Affiliations:** 1 Division of Hematology and Oncology, Department of Internal Medicine, Chungnam National University Hospital, Daejeon 35015, Republic of Korea; sorakang0515@cnu.ac.kr (S.K.); bluedays13@gmail.com (W.S.); goldjs2323@naver.com (J.S.K.); iyoo23@naver.com (M.-W.L.); petrosong@naver.com (I.-C.S.); cymed@cnu.ac.kr (H.-J.L.); deogyeon@cnu.ac.kr (D.-Y.J.); 2 Department of Internal Medicine, Chungnam National University College of Medicine, Daejeon 35015, Republic of Korea; yeeuni220@cnuh.co.kr; 3 Department of Bioengineering, University of California, Los Angeles, Los Angeles, CA 90095, USA; harinsim@g.ucla.edu (H.S.); jop@ucla.edu (J.O.P.); 4 Department of Chemical and Biomolecular Engineering, University of California, Los Angeles, Los Angeles, CA 90095, USA; samokeeffe@ucla.edu; 5 Division of Endocrinology, Department of Internal Medicine, Chungnam National University Hospital, Daejeon 35015, Republic of Korea; 6 Laboratory of Endocrinology and Immune System, Chungnam National University College of Medicine, Daejeon 35015, Republic of Korea

**Keywords:** sarcopenia, oral nutritional supplements, metabolites, microbiome, cancer cachexia, single-arm pilot study

## Abstract

Background/Objectives: Cancer cachexia is a multifactorial syndrome characterized by involuntary weight loss and muscle wasting, leading to impaired quality of life and poor clinical outcomes. Although oral nutritional supplements (ONS) are recommended to support inadequate oral intake during chemotherapy, their effects on underlying metabolic alterations and gut microbiome composition, particularly across different stages of cachexia remain unclear. This single-arm pilot study aimed to evaluate the feasibility and metabolic effects of an 8-week ONS intervention in patients with cancer cachexia undergoing chemotherapy. Methods: This study was conducted at the Chungnam National University Hospital, Daejeon, Republic of Korea between January 2023 and October 2023. The primary endpoints were feasibility outcomes, including adherence, tolerability, attrition rate, and ONS-related adverse events. Secondary outcomes included body composition, physical performance, biochemical markers, quality of life, plasma GDF-15 levels, serum metabolomics, and gut microbiome composition. Assessment of secondary outcomes and multi-omics profiling was performed at baseline and after 8 weeks. Patients were stratified into severe and non-severe cachexia groups and analyzed. Results: A total of 10 patients (median age 65 years, range 42–76) participated. Primary cancer types included cholangiocarcinoma (*n* = 4), colorectal (*n* = 4), and gallbladder cancer (*n* = 2). Adherence was 82%, with excellent tolerability and no ONS-related adverse events. Body composition, quality of life, and gut microbiome showed no significant changes. Hand-grip strength and walking-speed were slightly improved after 8 weeks intervention (*p* = 0.014 for hand-grip strength; *p* = 0.021 for walking-speed, Wilcoxon signed-rank test) in overall cohort. Metabolomics identified 10 metabolites, predominantly fatty acids, with significant between-group differential responses (*p* < 0.05, Mann–Whitney U test). Non-severe cachexia patients showed reductions in circulating fatty acids following ONS, consistent with attenuated lipolysis and reduced endogenous fat mobilization, whereas severe cachexia patients demonstrated increases, suggesting limited metabolic responsiveness to nutritional intervention. Fatty acid metabolism emerged as the predominant discriminatory pathway. Conclusions: This study showed the feasibility of integrating ONS with multi-omics profiling. Our findings suggest that metabolic alterations might precede clinically detectable changes, potentially providing a rationale for early intervention. Specifically, certain fatty acids were identified as candidate biomarkers that warrant further validation in larger cohorts.

## 1. Introduction

Cancer cachexia is a multifactorial syndrome characterized by substantial weight loss and skeletal muscle depletion, defined as >5% weight loss over the preceding 6 months or 2–5% weight loss in patients with a body mass index (BMI) < 20 kg/m^2^ or sarcopenia [1]. Approximately half of patients with advanced cancer experience cachexia [2], which significantly impairs their quality of life (QoL) through increased fatigue and functional decline [3]. More critically, cancer cachexia is associated with increased treatment-related toxicity, reduced chemotherapy dose intensity, decreased response rates, and ultimately poor overall survival [3,4,5,6,7]. Therefore, early detection and proactive management of cancer cachexia are essential for optimal patient care. Indeed, patients with advanced cancer express a significant perceived need for specialized nutritional support, particularly as their physical decline becomes apparent, highlighting the clinical necessity of addressing cachexia from the patient’s perspective [8]. International guidelines from ESPEN, ESMO, and ASCO consistently recommend early nutritional screening and intervention in cancer patients at risk of cachexia [2,9,10]. Recent clinical guidelines further emphasize a multidisciplinary approach integrating nutritional, pharmacological, and physical interventions tailored to cachexia stage [11].

The pathophysiology of cancer cachexia is inherently complex and distinct from simple malnutrition. It involves tumor-induced hypermetabolism affecting lipid, carbohydrate, and especially amino-acid metabolism, driven by an abnormal host response and systemic inflammation [12]. These alterations elevate resting energy expenditure and shift the body toward a negative energy balance, leading to progressive weight loss in the absence of adequate energy intake [12,13]. Recent multi-omics profiling has revealed that cancer cachexia involves a spatio-temporally coordinated metabolic reprograming across multiple tissues, characterized by alterations in amino acid, lipid, and one-carbon metabolism [14]. Primary anorexia is prevalent among cancer patients, while secondary factors—including treatment-related side effects such as nausea, vomiting, dysgeusia, and dysphagia—further compromise oral intake [12]. This reduced intake accelerates the progression of cachexia, creating a vicious circle of metabolic decay and physical wasting [13].

In this context, multimodal nutritional support has emerged as a cornerstone of cancer care to mitigate progressive wasting. This approach integrates dietary counseling, the management of symptoms that inhibit oral intake, and the administration of oral nutritional supplements (ONS) for malnourished patients capable of volitional intake [15,16]. ONS are specifically formulated to provide balanced energy and protein to bridge the gap between actual and required intake. A systematic meta-analysis indicated that nutritional interventions yield an overall benefit in body weight response during chemo(radio)therapy [17]. In particular, ONS enriched with *n*-3 polyunsaturated fatty acids (PUFAs), such as eicosapentaenoic acid (EPA), demonstrated significant improvements in body weight, the attenuation of lean body mass loss, and enhancement of certain QoL domains [17,18,19,20,21]. Beyond providing calories, nutritional intervention—particularly via multinutrient ONS—is increasingly recognized as a metabolic modulator, mitigating the systemic hypermetabolism by modulating inflammatory pathways and promoting protein synthesis [19,22,23,24]. However, a recent systematic review highlighted that despite some positive findings with specific supplements (EPA, β-HMB), the overall evidence for nutritional interventions in cancer cachexia remains inconclusive, with many trials failing to demonstrate significant benefits [25]. This underscores the need for early intervention and biomarker-guided patient selection.

The therapeutic potential of these nutritional interventions may, in part, be attributed to the modulation of the gut environment, alongside systemic anti-inflammatory effects. Emerging evidence suggests that gut microbiome plays a pivotal role in sustaining the systemic inflammation and metabolic alterations underlying cancer cachexia, similar to its involvement in other metabolic diseases, such as obesity and diabetes [26,27,28]. Specifically, microbiome dysbiosis compromises intestinal integrity, leading to increased intestinal permeability and subsequent gut barrier dysfunction. This phenomenon allows for the systemic translocation of endotoxins and bacterial products from the intestinal lumen, subsequently triggering a chronic host immune–inflammatory response and may contribute to the metabolic derangements associated with cachexia [29].

Beyond barrier dysfunction, gut microbiota also influences the production of critical bacterial metabolites, such as short-chain fatty acids (SCFAs) which possess potent anti-inflammatory properties. Recently, Ubachs et al. reported that SCFA levels were decreased in the patients with cancer cachexia compared to healthy controls, suggesting link between microbiome dysbiosis and decreased SCFA production [30]. Elucidating these intricate microbial and metabolic alterations is essential for developing targeted and personalized interventions. In this context, understanding how nutritional interventions, including ONS, influence the complex interplay between host metabolism and the gut microbiota is essential [28,31]. Such insights will provide a biological basis for optimizing therapeutic strategies that go beyond simple caloric supplementation to actively counteract the systemic drivers of cachexia [28,31,32].

However, significant knowledge gaps remain. First, while the effects of ONS on nutritional status and body composition have been investigated, their impact on metabolic profiles and gut microbiome composition—key factors in cachexia pathophysiology—remains largely unexplored. Second, it is unclear whether the efficacy of ONS differs according to cachexia severity. To address these gaps, we conducted this pilot study to evaluate the feasibility and safety of an 8-week ONS intervention in patients with cancer cachexia using multi-omics profiling, stratified into severe and non-severe groups. We assessed multiple outcomes, including body composition, dietary intake, physical performance, health-related QoL, biochemical parameters, metabolomic profiles, and gut microbiome composition at baseline and after 8 weeks of ONS supplementation.

## 2. Materials and Methods

### 2.1. Study Population

This prospective, single-arm pilot study was conducted at the Chungnam National University Hospital, Daejeon, Republic of Korea. Patients with cancer cachexia scheduled to initiate their first cytotoxic chemotherapy were recruited between January 2023 and October 2023. Potential participants were primarily identified by attending medical oncologists during routine outpatient consultations based on the clinical definition of cachexia. Following this initial identification, patients were referred to the research team, where a clinical research coordinator re-evaluated them against the specific inclusion and exclusion criteria. Final enrollment was confirmed only after the verification of eligibility and the acquisition of written informed consent. The inclusion criteria were: (1) histologically confirmed solid cancer requiring adjuvant or palliative chemotherapy and initiating chemotherapy for the first time; (2) presence of cachexia, defined as >5% unexplained weight loss within the previous 6 months before screening visit or consultation with medical oncologists; (3) age ≥ 19 years; (4) Eastern cooperative oncology group performance status 0–2; and (5) life expectancy ≥3 months as defined by investigators. Patients with known brain metastasis, malignant bowel obstruction, malignant ascites requiring paracentesis, uncontrolled infection, congestive heart failure, uncontrolled angina pectoris, cardiac arrhythmia, or psychiatric illness limiting study compliance were excluded. This study was approved by the Institutional Review Board (IRB) of the Chungnam National University Hospital (IRB number: 2022-05-088) and was conducted according to the principles of the Declaration of Helsinki. Written informed consent was obtained from all participants prior to enrollment.

### 2.2. Study Design and Intervention

Given the exploratory nature of this pilot study, a formal sample size calculation was not performed. Twelve patients were enrolled to evaluate study feasibility. Among them, one died before initiating the study protocol, and another withdrew consent after the first visit. Thus, ten patients completed the study protocol and were included in the analysis. The study flowchart is shown in Figure S1. To explore potential differences in ONS effects across cachexia severity, patients were stratified into severe and non-severe cachexia groups using phenotypic criteria consistent with the Global Leadership Initiative on Malnutrition framework [33]. Severe malnutrition (Stage 2) was defined by meeting at least one criterion: weight loss > 10% within the past 6 months (or >20% beyond 6 months), BMI < 18.5 kg/m^2^ if <70 years (or <20 kg/m^2^ if >70 years), or a severe deficit in muscle mass. Patients 1, 2, 3, 5, and 7 were classified as the severe group. The remaining patients (6, 8, 10, 11, and 12), who met at least one criterion for Stage 1/Moderate Malnutrition but not for Stage 2, were assigned to the non-severe group. All patients received ONS (provided by Maeil Dairies Co., Ltd., [Seoul, Republic of Korea]) at 200 mL twice daily for 8 weeks. Each 200 mL serving provided 200 kcal, 22.5 g carbohydrates, 7.5 g fat, 12.5 g protein, 4.2 g dietary fiber, and 135 mg *n*-3 PUFAs (docosahexaenoic acid and EPA). Detailed nutritional composition is provided in Table S1. Patient adherence was monitored through daily logs and weekly contact with the research team. Adherence rate was calculated as the percentage of prescribed supplements actually consumed.

**Figure 1:**
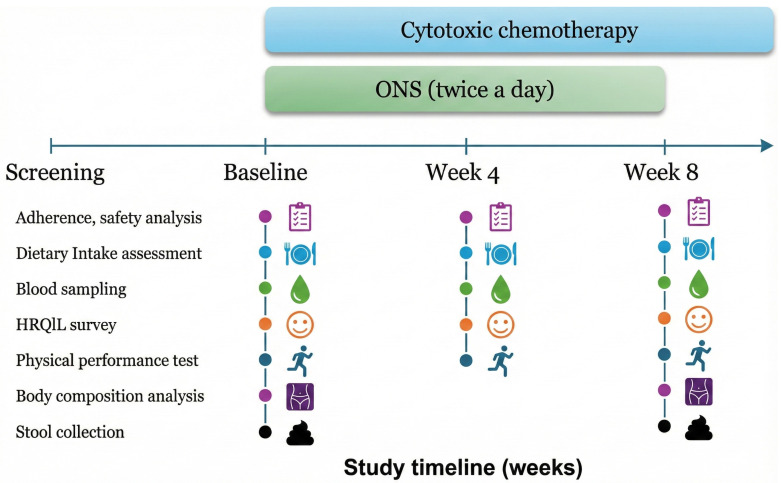
Study procedures and assessment schedule.

Before study initiation, comprehensive baseline assessments were performed, including blood sampling, stool collection, and dietary habit assessments via 24 h dietary recalls. We also measured mid-calf circumference and evaluated body composition using dual-energy X-ray absorptiometry (DXA). Physical performance was assessed through 4 m walk (4MW) and handgrip strength tests, while health-related QoL was surveyed using the European Organization for Research and Treatment of Cancer Quality of Life Questionnaire Core 30 (EORTC QLQ-C30). Throughout the study period, these assessments were carried out regularly every 4 weeks, with the exception of stool collection and DXA analysis, which were conducted only at baseline and at the 8-week follow-up (Figure 1).

During the study period, participants underwent systemic chemotherapy according to their treatment schedule. Adverse events were systemically recorded during clinic visits by attending physicians and were graded according to Common Terminology Criteria for Adverse Events, version 5.0 [34].

### 2.3. Study Endpoints

The primary endpoint was the feasibility of conducting ONS intervention integrated with multi-omics profiling in patients with cancer cachexia. Feasibility endpoints included tolerance to ONS, adherence to the 8-week ONS regimen, attrition rate, and ONS-related adverse events. Attrition rate was calculated by dividing the number of patients who did not complete the 8-week intervention by the total number of enrolled participants. ONS-specific adverse events were defined as gastrointestinal complications (e.g., nausea, vomiting, diarrhea, and abdominal bloating) occurring after ONS consumption, as well as any intolerance to the flavor or texture of the supplement.

Secondary endpoints included changes in body composition, physical function, biochemical markers (including Growth Differentiation Factor 15 [GDF-15]), and health-related QoL following ONS supplementation. Further, we evaluated changes in serum metabolite profiles and gut microbiome composition. All secondary endpoints were analyzed both for the overall cohort and stratified by cachexia severity (severe vs. non-severe) to explore potential differential response to ONS intervention across cachexia severity.

### 2.4. Assessments and Evaluations

#### 2.4.1. Dietary Intake

Dietary intake was assessed using the 24 h dietary recall method, which involves comprehensive documentation of all food and beverage consumption. Patients were requested to maintain a daily food diary throughout the study period, which was submitted at each clinic visit. Based on these diaries, energy and macronutrient intakes (carbohydrates, fats, and proteins) were calculated using the 2023 version of the National Food Nutrient Database provided by the Korean Ministry of Food and Drug Safety [35]. Data integration and total intake calculations for each participant were performed using Microsoft Excel (version 2016; Microsoft Corp., Redmond, WA, USA).

#### 2.4.2. Biochemical

Serum biochemical parameters were evaluated through blood sampling. These included complete blood cell counts (white blood cells, hemoglobin, platelets, neutrophils, and lymphocytes), inflammatory markers (C-Reactive protein [CRP] and interleukin-6, NLR, and PLR), nutritional status markers (total protein and albumin). All biochemical analyses were performed by the Department of Laboratory Medicine at Chungnam National University Hospital. Parameters were measured using standardized automated clinical chemistry and hematology analyzers according to the laboratory’s certified protocols. The analytical methods employed followed the specific enzymatic and immunoassay principles validated for clinical diagnostics.

#### 2.4.3. Anthropometric Measurements and Body Composition Analysis

Mid-calf circumference was measured at the widest point of the right calf with participants in an upright posture and equal weight distribution on both feet. Body composition was assessed using DXA (Horizon W, Hologic Inc., Bedford, MA, USA), providing comprehensive measurements of body weight, lean mass of each limb and trunk, bone mineral content, and fat mass distribution. Fat-free mass was calculated by subtracting fat mass from body weight. The lean body mass index (LMI), appendicular LMI, and fat-free mass index were calculated by dividing the respective mass measurements by height squared (kg/m^2^).

#### 2.4.4. Physical Performance

Physical function was evaluated using the 4MW and handgrip strength tests. For the 4MW test, participants walked at their usual pace along a 6 m path consisting of a 1 m acceleration zone, 4 m test zone, and 1 m deceleration zone. The test was performed twice, and the lower value was recorded. Handgrip strength was measured using a Smedley-type dynamometer (Takei T.K.K.5401 GRIP-D, Takei Scientific Instruments Co., Ltd., Tokyo, Japan). Participants held the dynamometer in their dominant hand with the arm at a right angle and elbow close to the body. They were instructed to squeeze with maximum force for approximately 5 s. The test was performed twice, and the higher value was recorded.

#### 2.4.5. QoL

Health-related QoL was assessed using the EORTC QLQ-C30 version 3 in Korean. This instrument comprises five functional scales (physical, role, emotional, cognitive, and social functioning), three symptom scales (fatigue, nausea/vomiting, and pain), six single-item symptom measures (dyspnea, insomnia, appetite loss, constipation, diarrhea, and financial difficulties), and a global health status/QoL scale. All scores were linearly transformed to a 0–100 scale. Higher scores on functional scales and global QoL indicated better functioning, whereas higher scores on symptom scales reflected greater symptom burden.

#### 2.4.6. Serum Metabolomics Analysis

To capture a broad range of polar and semi-polar metabolites, including amino acids, organic acids, and lipid-related intermediates, metabolites were extracted by mixing 10 μL of serum with 130 μL of 80% methanol, followed by centrifugation at 14,000× *g* for 10 min at 4 °C. Supernatants were transferred to high-performance liquid chromatography (HPLC) vials for analysis. Samples were analyzed using ultra-HPLC (Vanquish Duo UHPLC, Thermo Fisher Scientific, Waltham, MA, USA) coupled to an Orbitrap mass spectrometer (Q Exactive Plus, Thermo Fisher Scientific, Waltham, MA, USA). Liquid chromatography separation was performed on a hydrophilic interaction chromatography column (XBridge BEH Amide XP Column, 150 mm × 2.1 mm, 2.5-μm particle size; Waters Corp., Milford, MA, USA) using a gradient of solvent A (95:5 water/acetonitrile with 20 mM ammonium acetate and 20 mM ammonium hydroxide, pH 9.4) and solvent B (acetonitrile) [36]. The mass spectrometer was operated in both negative and positive ion modes at a resolution of 140,000 (at *m*/*z* 200), with a scan range of *m*/*z* 60–2000. Data was collected using Xcalibur software (version 4.3, Thermo Fisher Scientific, Waltham, MA, USA). Mass spectra and chromatograms were identified and integrated using metabolomic analysis and a visualization engine [37] with retention times determined by authenticated standards.

#### 2.4.7. Measurement of Plasma GDF-15 in Humans

Experiments were conducted with 50 plasma samples from 10 patients. The plasma samples were centrifuged immediately after collection and stored at −80 °C until analysis. Quantitative determination of GDF-15 in patient blood plasma samples was performed in triplicate, following the manufacturer’s protocol for the Human GDF-15 ELISA Kit (Human GDF-15 ELISA, R&D Systems, Abingdon, UK).

#### 2.4.8. Stool Sample Collections and Gut Microbiome Analysis

Fecal samples were collected from patients and promptly stored at −80 °C until further processing. For the analysis of gut microbiome profiles, total genomic DNA was extracted using a QIAamp Power Fecal Pro DNA Kit (Qiagen, Hilden, Germany). The quantity and quality of extracted DNA were measured using a Qubit Flex Fluorometer (Thermo Fisher Scientific, Waltham, MA, USA) and agarose gel electrophoresis, respectively. Targeted 16S sequencing libraries were prepared according to the 16S Metagenomics Sequencing Library Preparation protocol using the Illumina Nextera XT index and sequencing adapters (Illumina, San Diego, CA, USA). The V3–V4 hypervariable regions of the bacterial 16S rRNA were amplified using eight unique base pair barcodes and sequenced on an Illumina MiSeq system (Illumina, San Diego, CA, USA) according to the standard protocol [38]. Raw reads were analyzed using the QIIME2 pipeline (version 2023.9) [39]. Sequences were quality filtered and clustered into operational taxonomic units (OTUs) at 97% sequence identity, according to the SILVA 132 database [40]. OTUs were identified at the phylum and genus levels.

R-based packages, “microeco”, were used for microbiome analysis, and α-diversity was estimated using the Chao1 index and Shannon index. β-diversity was used to determine the differences between groups (baseline vs. 8 weeks). It was estimated using the Bray–Curtis distance and visualized using a principal coordinate analysis (PcoA) plot. Linear discriminant analysis (LDA) effect size (LefSe) was performed to identify significantly different taxa between the groups. An LDA score > 2.0 and false discovery rate (FDR)-corrected *p*-value < 0.1 were considered statistically significant.

### 2.5. Quantification and Statistical Analysis

Participant demographics were summarized using descriptive statistics. Continuous variables were compared between severe and non-severe groups using the Mann–Whitney U test, and within-participant changes from baseline to 4 and 8 weeks were assessed using the Wilcoxon signed-rank test. Categorical variables were compared using the χ^2^ test or Fisher’s exact test, as appropriate.

For metabolomic analysis, ion counts for each patient at each time point were normalized to baseline ion counts to calculate the relative change in metabolite levels over time. Differential metabolite changes (delta[Δ] = Week 8–Week 0) between severe and non-severe groups were compared using the Mann–Whitney U test. *p*-values were adjusted for multiple testing using the Benjamini–Hochberg FDR method. Effect sizes were calculated using Cohen’s *d*, with |*d*| > 0.8 considered a large effect. Partial least squares discriminant analysis (PLS-DA) was performed to assess overall metabolic separation between groups, with model validity evaluated using leave-one-out cross-validation and permutation testing (100 permutations). Variable Importance in Projection (VIP) scores were calculated to identify key discriminatory metabolites. Pathway enrichment analysis was performed using over-representation analysis with a hypergeometric test. Metabolites showing significant changes (FDR < 0.1) between severe and non-severe cachexia phenotypes over the 8-week intervention period were mapped to Kyoto Encyclopedia of Genes and Genomes (KEGG) pathways. Enrichment was quantified as the ratio of observed to expected proportion of changed metabolites. Other metabolomic analyses were performed using the MetaboAnalystR (version 6.0) [41] and “mixOmics” packages in R.

For gut microbiome analysis, α-diversity indices were compared between groups at each time point using the Mann–Whitney U test, and β-diversity was assessed using Permutational multivariate analysis of variance (PERMANOVA). All statistical tests were two-sided with α = 0.05, except for LEfSe analysis, which used an FDR threshold of 0.1. Analyses were performed using R version 4.2.2 (R Foundation for Statistical Computing, Vienna, Austria).

## 3. Results

### 3.1. Baseline Characteristics of Included Patients

Table 1 summarizes the baseline characteristics of the included patients. The median age was 65 years, and 70% of patients were male. Regarding cancer types, four patients had cholangiocarcinoma, four had colorectal cancer, and two had gallbladder cancer. Six patients received palliative chemotherapy, and four received adjuvant chemotherapy. When comparing the severe and non-severe groups, the severe group had lower BMI, lower pre-diagnosis body weight, and lower body weight at diagnosis, although these differences were not significant. No significant between-group differences were observed in other baseline characteristics. The detailed summary of the included patients is summarized in Table S2.

### 3.2. Primary Endpoints: Adherence and Tolerance to the ONS Regimen

All feasibility endpoints were successfully met. Among the 12 patients who initially enrolled, 10 completed the 8-week study period (attrition rate 16%), achieving 82% adherence to the ONS regimen (Table S2). Table 2 summarizes the adverse events that occurred during the study period. The most common adverse event was fatigue (40%), followed by anemia (30%). Most adverse events were grade 1 or 2 (82%), with six classified as grade 3. No grade 4 or 5 events occurred. All adverse events were attributed to cytotoxic chemotherapy, with no significant ONS-related adverse events reported, demonstrating excellent tolerance.

Serial specimen collection and assessments were successfully completed according to protocol. Blood and stool samples for metabolomics and microbiome analysis, respectively, were obtained from all patients at scheduled time points. Similarly, body composition analysis, physical performance tests, and QoL questionnaires were completed at all scheduled visits, achieving 100% completion rates for all study procedures.

### 3.3. Secondary Endpoints: Dietary Energy Intakes

Figure S2a–d summarize energy intake during the study period. At baseline, energy intake was higher in the severe group than in the non-severe group (Figure S2a, 1951 vs. 1847 kcal). After 4 weeks of ONS supplementation, total energy intake increased in both groups compared with baseline, reflecting ONS consumption. However, the increase was greater in the non-severe group, reversing the baseline pattern; at week 4, total energy intake was higher in the non-severe group (2197 vs. 2069 kcal). After 8 weeks, total energy intake decreased in both groups, reflecting reduced dietary intake owing to cumulative chemotherapy-related side effects. Regarding macronutrient composition, carbohydrate and protein intake showed similar trends to total energy intake (Figure S2b,c). Fat intake decreased in the severe group throughout the study period despite ONS supplementation. In the non-severe group, fat intake followed trends similar to those of other macronutrients (Figure S2d).

### 3.4. Secondary Endpoints: Body Composition Analysis by DXA

Table 3 presents body composition changes after 8 weeks of ONS administration. No significant differences were observed in body weight, lean body mass, or fat mass (all *p* > 0.05). We additionally conducted subgroup analysis according to cachexia severity. At baseline, the severe group had lower body weight (52.4 vs. 64.2 kg), lean body mass (35.51 vs. 40.74 kg), and fat mass (13.72 vs. 21.1 kg) than the non-severe group, although the differences were not significant (Table S3). After 8 weeks, the severe group experienced decreases in body weight (52.40–52.04 kg) primarily attributable to a decrease in fat mass (13.72–13.15 kg). Conversely, the non-severe group showed increases in body weight (64.20–65.48 kg), lean body mass (40.74–40.97 kg), and fat mass (21.10–22.10 kg).

### 3.5. Secondary Endpoints: Mid-Calf Circumferences & Physical Performance

**Figure 2:**
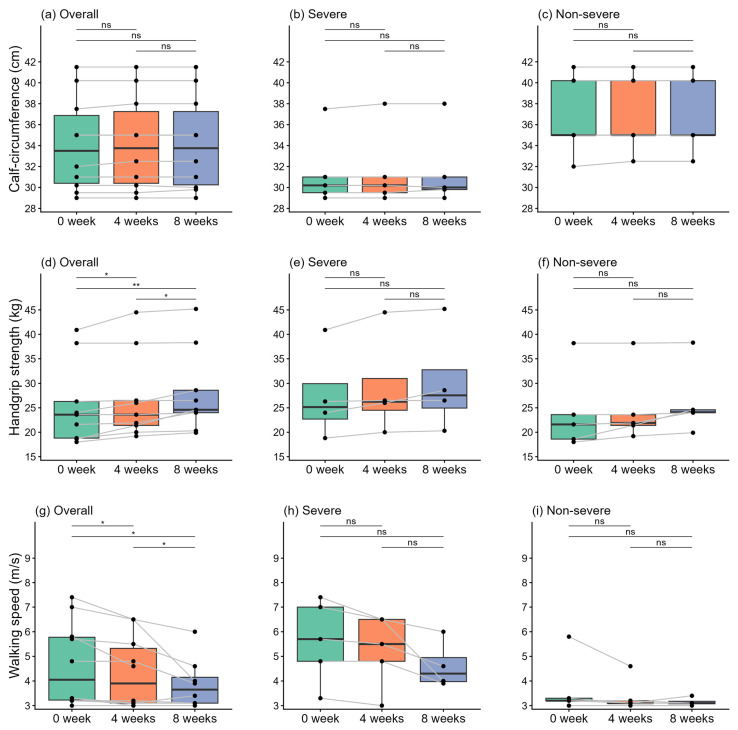
Changes in mid-calf circumference and physical performance tests. (**a**–**c**) Mid-calf circumference in (**a**) all participants, (**b**) severe group, and (**c**) non-severe group. (**d**–**f**) Hand grip strength in (**d**) all participants, (**e**) severe group, and (**f**) non-severe group. (**g**–**i**) Walking speed in (**g**) all participants, (**h**) severe group, and (**i**) non-severe group. Black dots represent individual data points, with gray lines indicating longitudinal changes for each subject. * *p* < 0.05, ** *p* < 0.01; ns, not significant.

Figure 2 shows changes in mid-calf circumference and physical performance tests overall and stratified by cachexia severity. At baseline, mid-calf circumference was lower in the severe group than in the non-severe group, reflecting the more pronounced sarcopenia in this group (Figure 2a). During the study period, mid-calf circumference showed no significant changes over time in either group (Figure 2a–c).

Regarding physical performance, hand grip strength slightly improved during the study period in the overall cohort (Figure 2d, *p* = 0.014); however, no significant changes were observed in either subgroup (Figure 2e,f). Similarly, walking speed in the overall cohort gradually improved, with a significant increase at week 8 compared with baseline (*p =* 0.021, Figure 2g). When analyzed by severity, the severe group demonstrated a trend toward improved walking speed similar to the overall cohort, although this improvement was not significant (Figure 2h). Conversely, walking speed remained relatively stable in the non-severe group across all time points (Figure 2i).

### 3.6. Secondary Endpoints: Biochemical Analysis, Including GDF-15

Table S4 and Figure S3 present the time-course changes of laboratory markers according to cachexia severity. At baseline, no significant differences were observed in most biochemical parameters between the severe and non-severe groups, likely owing to the small sample size. Nevertheless, the severe group demonstrated numerically higher inflammatory markers, including CRP (5.18 vs. 2.34 mg/dL), platelet count (383.20 vs. 291.00 × 10^3^/μL), and GDF-15 (978.05 vs. 919.68 pg/mL), suggesting a higher baseline inflammatory burden.

During the study periods, white blood cell counts and platelets decreased over time in both groups, reflecting myelosuppression due to chemotherapy toxicity. Regarding inflammatory markers, CRP levels in the severe group showed a transient increase in week 4 before declining to near-baseline levels at week 8. Conversely, the non-severe group maintained consistently low CRP levels throughout the study period. The neutrophil-to-lymphocyte ratio (NLR) and platelet-to-lymphocyte ratio (PLR) showed similar patterns to CRP levels. GDF-15, a biomarker associated with cancer cachexia, remained consistently elevated in the severe group throughout the study period (978.05 pg/mL at baseline to 1031.71 pg/mL at week 8), while showing a declining trend in the non-severe group (919.68 pg/mL to 667.95 pg/mL).

### 3.7. Secondary Endpoints: Health-Related QoL

Table S5 summarizes the changes in Health-related QoL measured by the EORTC QLQ-C30. At baseline, no significant between-group differences were observed. Functional scale scores remained relatively stable in both groups throughout the study period, with no significant between-group differences at any time point. Overall symptom burden also remained stable, with no significant between-group differences (all *p* > 0.05).

### 3.8. Secondary Endpoints: Changes in Serum Metabolite Levels

At baseline (week 0), metabolic profiling of 134 metabolites revealed minimal between-group differences (Figure S4a), except hyodeoxycholic acid, which was significantly higher in the severe group (Figure S4b, *p =* 0.0367).

**Figure 3:**
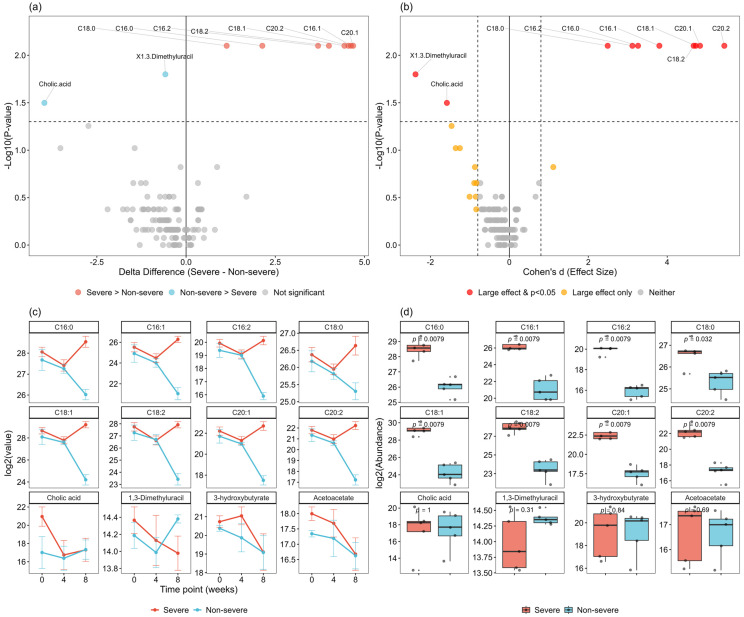
Results of serum metabolite analysis (**a**) Volcano plots showing metabolite differences between baseline and 8 weeks according to cachexia severity groups. Dashed lines indicate the thresholds for statistical significance (*p* < 0.05) and fold-change. Gray dots represent non-significant metabolites. (**b**) Effect size vs. statistical significance. The solid vertical line indicates an effect size of zero, and the dashed vertical lines represent the thresholds for a large effect size (*|Cohen’s d|* > 0.8). The horizontal dashed line indicates the threshold for statistical significance (*p* < 0.05) (**c**) Changes in significant metabolites during the study period according to cachexia severity groups. (**d**) Boxplots of significant metabolites at week 8 according to cachexia severity groups.

Following 8 weeks of ONS intervention, analysis of metabolite changes (Δ) identified 10 metabolites (7.5%) that differed significantly between groups (*p* < 0.05, Figure 3a). Although none survived stringent FDR correction at 0.1, eight metabolites (6.0%) remained significant at FDR < 0.2, suggesting meaningful biological differences. Effect size analysis demonstrated that 20 metabolites (14.9%) exhibited large effect sizes (|*d*| > 0.8), indicating potential biological relevance (Figure 3b).

Among the 10 significantly different metabolites, eight were identified as fatty acids, representing the predominant metabolic class affected by ONS in patients with severe cachexia: palmitic acid (C16:0), stearic acid (C18:0), palmitoleic acid (C16:1), oleic acid (C18:1), eicosenoic acid (C20:1), hexadecadienoic acid (C16:2), linoleic acid (C18:2), and eicosadienoic acid (C20:2) (Figure 3c,d). All eight fatty acids demonstrated large to very large effect sizes (|*d*| range: 2.1–4.8). Following ONS intervention, the non-severe cachexia group showed substantially greater decreases in these metabolites than the severe group, which exhibited substantial increases. However, ketone bodies (end products of fatty acid metabolism, including 3-hydroxybutyrate and acetoacetate) exhibited similar patterns in both groups, with slight increases at week 4 followed by a decrease at week 8. Unlike fatty acids, bile acid metabolites, such as cholic acid, decreased in the severe group, whereas the non-severe group showed stable levels (Figure 3c,d).

Amino acid metabolites, tricarboxylic acid (TCA) cycle intermediates (α-ketoglutarate, aconitate, citrate, fumarate, malate, and succinate), and glucose metabolism intermediates (D-glucose, methylglyoxal, and pyruvate) showed similar decreasing trends over the study period in both groups, with no significant between-group differences (all *p* > 0.05, Figure S5a for TCA cycle intermediates, Figure S5b for glucose metabolism intermediates).

**Figure 4:**
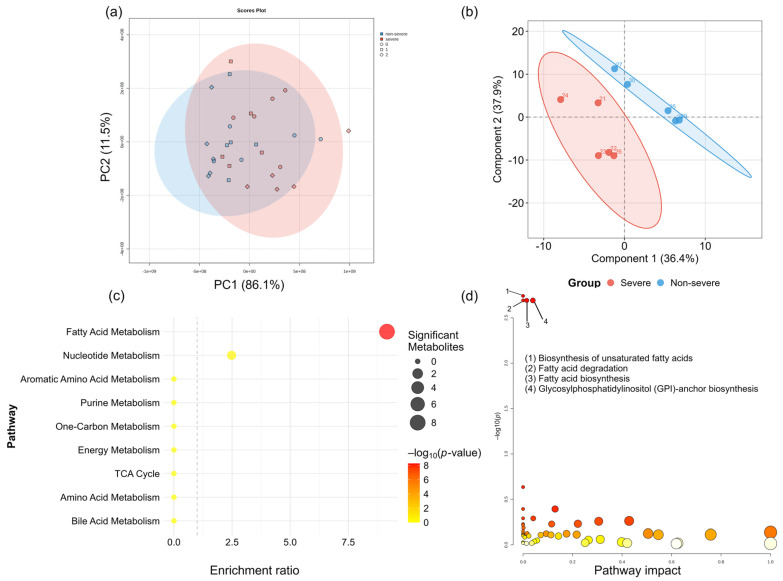
Serum metabolite analysis (**a**) Principal component analysis (PCA). (**b**) Partial least squares discriminant analysis (PLS-DA). The shaded areas in (**a**,**b**) represent the 95% confidence ellipses for each group, where red and blue indicate the severe and non-severe groups, respectively. (**c**) Pathway enrichment analysis performed using over-representation analysis based on delta metabolites. The dashed line at indicates the baseline where the enrichment ratio is equal to 1, representing no enrichment effect. Pathways located to the right of this line show a higher enrichment ratio than expected by chance. (**d**) Pathway enrichment analysis performed using MetaboAnalyst version 6.0.

We conducted multivariate analysis, including principal component analysis (PCA) and PLS-DA. Figure 4a shows the PCA results. PCA of metabolite profiles across all timepoints (0, 4, and 8 weeks) revealed significant between-group differences (PERMANOVA: *F* = 5.17, *R*^2^ = 0.156, *p* = 0.018 based on 999 permutations). The first two principal components explained 97.6% of the total variance (PC1: 86.1%, PC2: 11.5%). However, considerable overlap was observed between groups, with the group effect accounting for only 15.6% of the total metabolic variation.

Figure 4b shows the results of the PLS-DA model based on delta metabolite profiles. The PLS-DA model showed visual separation between the groups compared with PCA (Figure 4a). However, permutation testing did not identify statistical significance of this separation (*p* = 0.66, 100 permutations), likely attributable to the limited sample size (*n* = 5 per group) and high inter-individual variability. VIP analysis identified 31 metabolites with VIP > 1, predominantly fatty acids and bile acids (cholic acid), which were previously highlighted in the univariate analysis (Table S6).

Pathway enrichment analysis using over-representation analysis with a hypergeometric test on delta metabolites values revealed lipid metabolism enrichment, specifically fatty acid metabolism, among metabolites altered between severe and non-severe cachexia phenotypes (*p* < 1 × 10^−8^, enrichment ratio = 9.16). Of the 13 metabolites measured in the fatty acid metabolism pathway, eight were significantly altered (61.5%), including saturated (C16:0 and C18:0) and polyunsaturated (C18:2 and C20:2) fatty acids. Conversely, other major metabolic pathways, including amino acid metabolism and the TCA cycle, showed no significant enrichment (Figure 4c). Validation using quantitative enrichment analysis of 8-week endpoint metabolomic data in MetaboAnalystR similarly identified fatty acid metabolism as the predominant enriched pathway, consistent with the analysis based on delta metabolite profiles (Figure 4d).

Additionally, we performed correlation analyses between the observed metabolomic shifts and clinical outcomes (e.g., changes in body composition and physical performance); however, no statistically significant correlations were identified (Figure S6). 

### 3.9. Secondary Endpoints: Changes in Gut Microbiome Compositions

**Figure 5:**
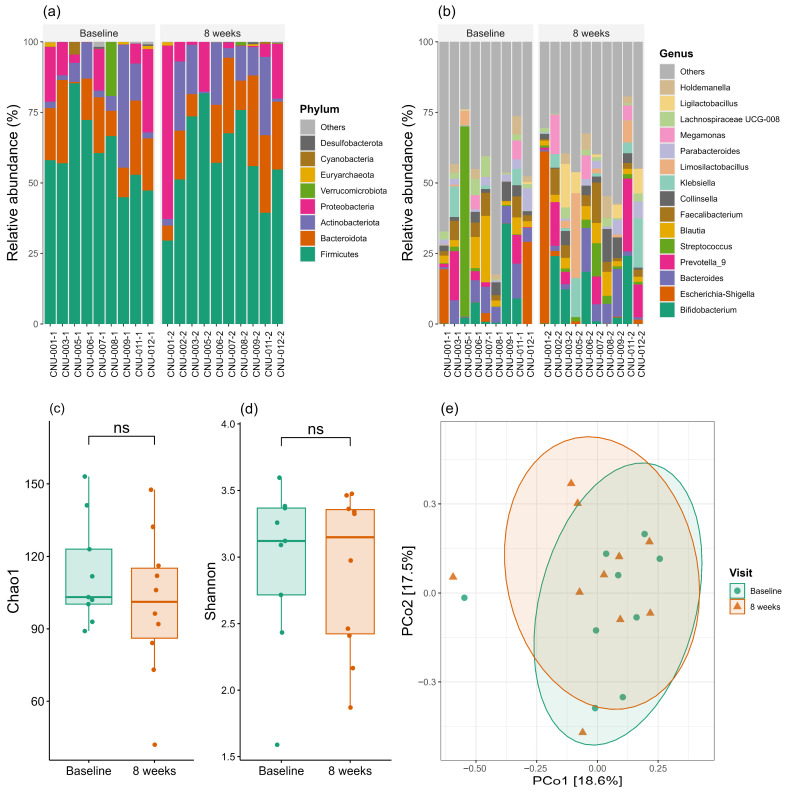
Gut microbiome profiles and α/β diversity analysis between baseline and after 8 weeks of ONS administration (**a**) Phylum-level taxonomic composition showing the relative abundance of the eight most abundant bacterial phyla at baseline and 8 weeks. (**b**) Genus-level taxonomic composition displaying the relative abundance of the fifteen most abundant bacterial genera. (**c**) Chao1 richness index. (**d**) Shannon diversity index. (**e**) Principal coordinate analysis (PCoA) plot based on Bray–Curtis dissimilarity showing β-diversity separation. ns, not significant.

Figure 5a,b compares microbiome profiles at baseline and after 8 weeks of ONS administration. At the phylum level (Figure 5a), *Firmicutes* were the most abundant, comprising approximately 40–70% of the total microbiota, followed by *Bacteroidota*, *Actinobacteriota*, and *Proteobacteria*. The relative abundance of *Firmicutes* remained relatively stable between baseline and week 8. At the genus level (Figure 5b), the microbiota displayed greater complexity and variability. The most abundant genera identified included *Bifidobacterium*, *Escherichia-Shigella*, *Prevotella_9*, *Bacteroides*, *Faecalibacterium*, *Collinsella*, *Klebsiella*, *Limosilactobacillus*, *Parabacteroides*, *Megamonas*, *Lachnospiraceae UCG-008*, *Ligadibacterium*, and *Holdemanella*. Relative abundances varied considerably among individuals, with some showing marked shifts between baseline and week 8.

α-diversity metrics demonstrated no significant difference between baseline and week 8 (Figure 5c,d), indicating that bacterial species richness and evenness were largely maintained throughout the study period, and ONS administration did not substantially alter the overall microbial diversity within individuals. Similarly, PCoA plot based on Bray–Curtis dissimilarity (Figure 5e) revealed considerable overlap between baseline and week 8, indicating that the overall community structure was largely preserved over time. LEfSE analysis identified no significantly different taxa between the two time points.

We conducted the same analysis according to cachexia severity. Similar to the overall population, no significant changes in microbiome profiles were observed after 8 weeks of ONS administration in both groups (Figure S7 [severe group] and Figure S8 [non-severe group]).

## 4. Discussion

This pilot study evaluated the feasibility of an 8-week ONS intervention with comprehensive multi-omics profiling in patients with cancer cachexia undergoing standard cytotoxic chemotherapy. Adherence to the ONS regimen was high (82%), with excellent tolerance and no significant ONS-related adverse events, confirming that nutritional supplementation can be safely implemented alongside chemotherapy. Serial collection of biological specimens and completion of physical performance assessments further support the practicality of conducting multi-modal cachexia research in routine clinical settings.

Despite meeting all feasible endpoints, most secondary endpoints—including body composition (except BMI), mid-calf circumference, biochemical markers, or health-related QoL—showed no significant changes following ONS supplementation. This limited impact on phenotypic endpoints emphasizes the inherent difficulty of reversing a multifactorial metabolic syndrome such as cancer cachexia using nutritional support alone. Nevertheless, significant improvements in physical performance, including handgrip strength and walking speed, suggest that nutritional supplementation may confer functional benefits independent of measurable changes in body composition.

Multi-omics profiling revealed differential biological responses to ONS according to cachexia severity. GDF-15 is a stress-induced cytokine implicated in appetite suppression, lipolysis, and muscle wasting; its elevation is associated with weight loss and poor survival in cancer patients [42,43]. The clinical significance of GDF-15 has been further underscored by recent clinical trials of Ponsegromab, which demonstrated that inhibiting GDF-15 can improve body weight and physical activity in cachectic patients [44]. In the present study, GDF-15 levels remained persistently elevated in the severe group, whereas a downward trend was observed in the non-severe group. While these divergent trajectories did not reach statistical significance, they may suggest that metabolic plasticity and responsiveness to nutritional intervention are better preserved in the earlier stages of cachexia. These findings highlight the potential of GDF-15 as a candidate indicator of metabolic responsiveness, although further validation in larger, sufficiently powered cohorts is warranted.

Metabolomics analyses further support this concept of stage-dependent metabolic plasticity. Following ONS intervention, the non-severe group exhibited substantial reductions in circulating fatty acids, whereas the severe group showed paradoxical increase. Crucially, eight of ten discriminatory metabolites were fatty acids, indicating the central role of dysregulated lipid metabolism in cachexia [45]. The remaining two metabolites were ketone bodies, which demonstrated comparable trajectories in both groups, suggesting that the observed differences predominantly reflect altered lipolysis rather than changes in fatty acid oxidation [31,46]. Additionally, pathway enrichment analysis identified fatty acid metabolism as the primary discriminatory pathway, aligning with accelerated adipose tissue lipolysis which is a hallmark of cachexia [47,48,49].

As carbohydrate-containing ONS can suppress lipolysis through insulin-mediated inhibition of hormone-sensitive lipase, the observed reduction in fatty acids among the non-severe group likely reflects improved metabolic plasticity and reduced reliance on endogenous fat mobilization. Enhanced lipolysis leads to excessive release of circulating fatty acids, which can induce lipotoxicity and contribute to muscle wasting through adipose–muscle crosstalk [45,50,51]. Thus, inhibiting lipolysis has emerged as a potential therapeutic strategy in cancer cachexia management, including interventions targeting GDF-15 and triglyceride lipase [50,52,53]. Conversely, the increase in circulating fatty acids in the severe group may reflect a state of advanced metabolic dysregulation, in which nutritional supplements might be insufficient to attenuate accelerated lipolysis potentially driven by systemic inflammation, mitochondrial dysfunction, and impaired re-esterification capacity. While direct functional validation of these shifts was not within the scope of this pilot study, our findings provide a hypothesis for further investigation into the mechanistic drivers of metabolic refractory states in advanced cachexia.

Despite distinct metabolic responses, we observed no significant changes in routine clinical markers (CRP, albumin) or phenotypic endpoints, including body composition and mid-calf circumference. These findings are consistent with prior randomized controlled trials (RCTs) showing limited clinical efficacy of ONS monotherapy [25,54]. Collectively, these data indicate that metabolomic alterations may occur prior to measurable phenotypic recovery. Consequently, metabolomics may serve as an early marker for treatment responsiveness, identifying patients who retain metabolic plasticity. Further studies with longer follow-ups and sensitive imaging tools are needed to confirm whether these early metabolic shifts translate into clinical benefits. Additionally, given that ONS alone might be insufficient to achieve meaningful clinical efficacy [25,54], thus multimodal interventions—integrating enhanced nutritional support with physical rehabilitation and pharmacological approaches—are likely required to achieve improvements in patient outcomes.

In contrast to metabolomics findings, gut microbiome composition remained largely unchanged after 8 weeks of ONS administration. Neither α- nor β-diversity metrics showed meaningful alteration, and no taxonomic features were differentially abundant, highlighting the challenge of modulating dysbiosis through nutritional interventions alone. Microbiome variability and the small sample size may have further limited detection of meaningful changes, underscoring the complexity of microbiome-targeted strategies in cancer cachexia [11].

### 4.1. Limitations of the Study

This study has several limitations. First, the small sample size (*n* = 10) substantially limits statistical power and generalizability. As an exploratory pilot study, no priori power calculations or formal sample size calculations were performed, restricting our ability to draw definitive conclusions regarding ONS efficacy. The limited sample size particularly affects our subgroup comparisons between severe and non-severe cachexia, which may be underpowered to detect true differences. Patient heterogeneity, including variations in tumor types, clinical stages, and chemotherapy regimens, further introduces substantial variability that may confound the interpretation of our results. Additionally, several potentially confounding factors were not strictly controlled in this pilot study, such as baseline inflammatory states, the use of corticosteroids, and total dietary intake. The lack of objective monitoring for physical activity levels also limits our ability to fully differentiate the impact of ONS from other lifestyle factors. Second, this study was conducted at a single tertiary care institution without external validation cohorts, further limiting the generalizability of the findings to other clinical settings or patient populations. The absence of a control group and the relatively short 8-week intervention period may also have precluded meaningful changes in secondary outcomes. Third, the observed metabolomic alterations did not show direct statistical correlations with phenotypic clinical outcomes, which may be due to the small sample size and the short follow-up period of this pilot study. Fourth, while our study tracked changes over an 8-week period, the small sample size precluded a robust longitudinal statistical analysis, such as linear mixed-effects modeling. Consequently, these findings should be interpreted as preliminary and hypothesis-generating rather than as definitive evidence of ONS efficacy.

Despite these limitations, our study provides several important insights. It highlights the feasibility of integrating multi-omics profiling into cancer cachexia intervention trials, identifies fatty acid metabolism and GDF-15 as potential stage-specific biomarkers of metabolic responsiveness to nutrition therapy, and suggests that early cachexia may represent a unique “window of opportunity” during which metabolic plasticity is still preserved, and nutritional interventions may be more effective. These insights will serve as a foundation for designing future large-scale, multi-center RCTs, with larger, more homogeneous patient cohorts, detailed metabolic profiling, sensitive body composition assessment methods, and inclusion of control groups to definitively establish the role of early nutritional intervention in managing cancer cachexia across different severity stages.

### 4.2. Future Perspectives

Based on our exploratory findings, several directions for future research are warranted. First, these results should be validated in larger, multicenter RCTs with adequate statistical power and stratification by cachexia severity. Second, longer follow-up periods are essential to determine whether early metabolomic shifts eventually translate into meaningful clinical outcomes, such as improved body composition or survival. Third, comprehensive phenotyping, incorporating diverse inflammatory and hormonal markers, will help clarify the underlying mechanisms of stage-dependent metabolic plasticity. Furthermore, the use of advanced imaging techniques may improve the detection of subtle changes in body composition that were not captured by conventional measures. Future studies should also explore multidisciplinary interventions that combine optimized nutrition with pharmacological therapies and structured exercise programs. Refinement of ONS formulations, potentially enriched with specific metabolic modulators, may further enhance therapeutic efficacy. Finally, circulating fatty acids and GDF-15 require external validation as predictive biomarkers to support the development of personalized, stage-specific nutritional strategies in the management of cancer cachexia.

## 5. Conclusions

In conclusion, this pilot study demonstrated the feasibility and excellent tolerability of an 8-week ONS intervention integrated with multi-omics profiling in patients with cancer cachexia. Although traditional clinical endpoints—including body composition and QoL—and gut microbiome profiles showed no significant changes, metabolomic analysis revealed preliminary evidence of stage-dependent metabolic responses. Specifically, a subset of metabolites, predominantly fatty acids, exhibited significant differential responses between groups. These findings highlight the potential of circulating fatty acids as candidate metabolic indicators for monitoring nutritional responsiveness and suggest the importance of early intervention, when metabolic plasticity may still be preserved. However, further validation through ROC analysis and larger-scale clinical correlation is required to establish their utility as clinical biomarkers.

Despite limitations in sample size and intervention duration, this study provides a foundational framework supporting multi-omics-integrated approaches and offers exploratory signals that warrant validation in larger, controlled, and stage-stratified clinical trials. Ultimately, our results emphasize the need for further investigation into stage-specific and metabolically informed nutritional strategies in cancer cachexia management.

## Figures and Tables

**Table 1:** Patient baseline characteristics.

Characteristic	Overall (*n* = 10)	Severe (*n* = 5)	Non-Severe (*n* = 5)	*p*-Value *
Body mass index, kg/m^2^ (SD)	22.2 (4.3)	20.3 (2.1)	24.1 (5.2)	0.2
Body weight before diagnosis, kg	67.9 (13.4)	62.8 (12.4)	73.0 (13.7)	0.2
Body weight at the time of diagnosis, kg	58.7 (11.9)	53.0 (8.0)	64.4 (13.1)	0.093
Age, median (range)	65.0 (42.0–76.0)	68.0 (42.0–74.0)	58.0 (54.0–76.0)	>0.9
Age < 65 years	5 (50%)	2 (40%)	3 (60%)	
Age ≥ 65 years	5 (50%)	3 (60%)	2 (40%)	
Sex				>0.9
Male	7 (70%)	3 (60%)	4 (80%)	
Female	3 (30%)	2 (40%)	1 (20%)	
ECOG performance score				0.4
0	8 (80%)	3 (60%)	5 (100%)	
1	2 (20%)	2 (40%)	0 (0%)	
Smoking				>0.9
Never-smoker	3 (30%)	1 (20%)	2 (40%)	
Ex-smoker	7 (70%)	4 (80%)	3 (60%)	
Alcohol consumption				>0.9
Never-drinker	2 (20%)	1 (20%)	1 (20%)	
Ex-drinker	8 (80%)	4 (40%)	4 (40%)	
Hypertension	3 (30%)	3 (60%)	0 (0%)	0.2
Diabetes mellitus	5 (50%)	4 (80%)	1 (20%)	0.2
Types of cancer				0.7
Cholangiocarcinoma	4 (40%)	3 (60%)	1 (20%)	
Colorectal cancer	4 (40%)	1 (20%)	3 (60%)	
Gallbladder cancer	2 (20%)	1 (20%)	1 (20%)	
Types of treatment				0.5
Adjuvant	4 (40%)	3 (60%)	1 (20%)	
Palliative	6 (60%)	2 (40%)	4 (80%)	

ECOG, Eastern Cooperative Oncology Group; SD, standard deviation. * χ^2^ test or Fisher’s exact test.

**Table 2:** Adverse events during the 8-week study period.

Adverse Event	Total *n* (%)	Grade 1	Grade 2	Grade 3
Abdominal pain	2 (20%)	1	0	1
Alanine Aminotransferase elevation	2 (20%)	2	0	0
Anemia	3 (30%)	1	2	0
Aspartate Aminotransferase elevation	2 (20%)	2	0	0
Chest Pain	1 (10%)	0	1	0
Constipation	1 (10%)	0	1	0
Cough	1 (10%)	1	0	0
Diarrhea	4 (40%)	0	3	1
Esophagitis	1 (10%)	1	0	0
Fatigue	4 (40%)	1	3	0
Fever	2 (20%)	0	1	1
Hand Foot Syndrome	1 (10%)	0	1	0
Mucositis	2 (20%)	0	1	1
Neutropenia	2 (20%)	0	0	2
Peripheral neuropathy	2 (20%)	1	1	0
Pruritus	1 (10%)	1	0	0
Sputum	1 (10%)	1	0	0
Thrombocytopenia	1 (10%)	1	0	0
Total number of adverse events		14 (41%)	14 (41%)	6 (17%)

**Table 3:** Changes in body Composition from baseline to week 8.

Characteristics	0 Week	8 Weeks	*p*-Value *
Body weight (kg)	58.30 (12.03)	58.76 (11.68)	0.55
Lean body mass (kg)	38.13 (5.45)	38.56 (5.71)	0.27
Bone mineral contents (kg)	1.95 (0.27)	1.93 (0.26)	0.28
Fat mass (kg)	17.41(9.85)	17.62 (9.54)	0.62
Fat-free mass (kg)	40.07 (5.60)	40.48 (5.85)	0.27
Fat-free mass index (kg/m^2^)	15.07 (1.51)	15.23 (1.64)	0.37
Lean body mass index (kg/m^2^)	14.42 (1.41)	14.60 (1.56)	0.12
Appendicular lean body mass index (kg/m^2^)	5.53 (0.72)	5.70 (0.92)	0.10

* Wilcoxon signed-rank test.

## Data Availability

The data are not publicly available due to ethical restrictions. The data presented in this study are available on request from the corresponding author.

## Supplementary Materials

The following supporting information can be downloaded at: https://www.mdpi.com/article/10.3390/nu18040597/s1, Figure S1: CONSORT diagram of this study; Figure S2: Changes in dietary intakes by cachexia severity group; Figure S3: Changes in biochemical markers by cachexia severity group; Figure S4: Results of serum metabolite analysis: (a) Volcano plots at baseline; (b) Levels of hyodeoxycholic acid according to cachexia severity group; Figure S5: Results of serum metabolite analysis: (a) Changes in TCA cycle intermediates by Cachexia Severity Group; (b) Glucose metabolism intermediates by Cachexia Severity Group; Figure S6. Correlation heatmap between serum metabolites and clinical parameters. The heatmap illustrates Spearman’s rank correlation coefficients between the top 50 serum metabolites (ranked by variance) and key clinical factors related to cancer cachexia. Red and blue colors represent positive and negative correlations, respectively, as indicated by the color scale bar. Numbers within the cells indicate the correlation coefficient (r). Clinical parameters include calf circumference (leg), handgrip strength (hand), walking speed, and biochemical markers (albumin, CRP, and IL-6). Figure S7: Gut Microbiome profiles and α/β diversity analysis between baseline and after 8 weeks of ONS administration in the severe group: (a) Phylum-level taxonomic composition showing the relative abundance of the eight most abundant bacterial phyla at baseline and after 8 weeks; (b) Genus-level taxonomic composition displaying the relative abundance of the fifteen most abundant bacterial genera; (c) Chao1 richness index; (d) Shannon diversity index; (e) Principal coordinate analysis (PCoA) plot based on Bray–Curtis dissimilarity; Figure S8: Gut microbiome profiles and α/β diversity analysis between baseline and after 8 weeks of ONS administration in the non-severe group: (a) Phylum-level taxonomic composition showing the relative abundance of the eight most abundant bacterial phyla at baseline and 8 weeks. (b) Genus-level taxonomic composition displaying the relative abundance of the fifteen most abundant bacterial genera; (c) Chao1 richness index; (d) Shannon diversity index; (e) Principal coordinate analysis (PCoA) plot based on Bray–Curtis dissimilarity; Table S1: Nutritional composition of oral nutrient supplements developed by Maeil Dairies Co., Ltd.; Table S2: Detailed information on the included patients; Table S3: Body composition analysis via DXA scan at baseline and 8 weeks according to cachexia severity; Table S4: Changes in biochemical markers at baseline, after 4 weeks, and 8 weeks according to cachexia severity; Table S5: Changes in quality of life and symptom scores (EORTC QLQ-C30) across treatment periods by cachexia severity group; Table S6: Variable Importance in Projection (VIP) scores of metabolites associated with differential metabolic response to ONS intervention in cancer cachexia (VIP > 1.0).

## Abbreviations

The following abbreviations are used in this manuscript:

4MW	4 m Walk test
ALMI	Appendicular Lean Body Mass Index
BMI	Body Mass Index
CRP	C-reactive Protein
CTCAE	Common Terminology Criteria for Adverse Events
DXA	Dual-energy X-ray Absorptiometry
ECOG	Eastern Cooperative Oncology Group
EPA	Eicosapentaenoic Acid
FDR	False Discovery Rate
FFMI	Fat-Free Mass Index
GDF-15	Growth Differentiation Factor 15
GLIM	Global Leadership Initiative on Malnutrition
IRB	Institutional Review Board
KEGG	Kyoto Encyclopedia of Genes and Genomes
LC-MS	Liquid Chromatography-Mass Spectrometry
LDA	Linear Discriminant Analysis
LEfSe	Linear Discriminant Analysis Effect Size
LMI	Lean Body Mass Index
NLR	Neutrophil-to-Lymphocyte Ratio
ONS	Oral Nutritional Supplements
OTUs	Operational Taxonomic Units
PCA	Principal Component Analysis
PCoA	Principal Coordinate Analysis
PERMANOVA	Permutational Multivariate Analysis of Variance
PLR	Platelet-to-Lymphocyte Ratio
PLS-DA	Partial Least Squares Discriminant Analysis
QoL	Quality of Life
TCA	Tricarboxylic Acid cycle
UHPLC	Ultra-High-Performance Liquid Chromatography
VIP	Variable Importance in Projection
