# Supplementary material for: Stage-Dependent Metabolic Responses to Oral Nutritional Supplementation in Cancer Cachexia: A Single-Arm Pilot Study
Source: Nutrients. 2026 Feb 11;18(4):597. doi: 10.3390/nu18040597 (PMC12943304; doi:10.3390/nu18040597)
Supplement: Supplementary file 1 [file nutrients-18-00597-s001.zip › nutrients-4126307-supplementary.pdf]

SUPPLEMENTAL INFORMATION

Table S1. Nutritional composition of oral nutrient supplements developed by Maeil Dairies Co., Ltd.. 1

Table S2. Detailed information on the included patients 3

Table S3. Body composition analysis via DXA scan at baseline and 8 weeks according to cachexia severity 5

Table S4. Changes in biochemical markers at baseline, after 4 weeks, and 8 weeks according to cachexia severity. 6

Table S5. Changes in quality of life and symptom scores (EORTC QLQ-C30) across treatment periods by cachexia severity group 8

Table S6. Variable Importance in Projection (VIP) scores of metabolites associated with differential metabolic response to ONS intervention in cancer cachexia (VIP > 1.0) 10

Figure S1. CONSORT diagram of this study. 13

Figure S2. Changes in dietary intakes by cachexia severity group. (a) Total energy intake (b) carbohydrate (c) protein, and (d) fat. 14

Figure S3. Changes in biochemical markers by cachexia severity group. 15

Figure S4. Results of serum metabolite analysis. (a) Volcano plots at baseline. (b) Levels of hyodeoxycholic acid according to cachexia severity group. 16

Figure S5. Results of serum metabolite analysis: (a) Changes in tricarboxylic acid cycle intermediates by Cachexia Severity Group; (b) Glucose metabolism intermediates by Cachexia Severity Group. 17

Figure S6. Gut Microbiome profiles and α/β diversity analysis between baseline and after 8 weeks of ONS administration in the severe group: (a) Phylum-level taxonomic composition showing the relative abundance of the eight most abundant bacterial phyla at baseline and after 8 weeks; (b) Genus-level taxonomic composition displaying the relative abundance of the fifteen most abundant bacterial genera; (c) Chao1 richness index; (d) Shannon diversity index; (e) Principal coordinate analysis (PCoA) plot based on Bray-Curtis dissimilarity. 19

Figure S7. Gut microbiome profiles and α/β diversity analysis between baseline and after 8 weeks of ONS administration in the non-severe group: (a) Phylum-level taxonomic composition showing the relative abundance of the eight most abundant bacterial phyla at baseline and 8 weeks; (b) Genus-level taxonomic composition displaying the relative abundance of the fifteen most abundant bacterial genera; (c) Chao1 richness index; (d) Shannon diversity index; (e) Principal coordinate analysis (PCoA) plot based on Bray-Curtis dissimilarity. 20

Table S1. Nutritional composition of oral nutrient supplements developed by Maeil Dairies Co., Ltd.

Components	Per 200 mL	Units
Calories	200	kcal
Sodium	200	mg
Carbohydrates	22.5	g
Sugar	11	g
Dietary fiber	4.2	g
Fat	7.5	g
Trans Fat	0	g
Saturated Fat	1	g
Cholesterol	0	mg
Protein	12.5	g
Vitamin A	200	μg RE
Vitamin D	2	Mg
Vitamin E	6	mgα-TE
Vitamin K	18	μg
Vitamin B1 (Thiamine)	0.36	mg
Vitamin B2 (Riboflavin)	0.4	mg
Vitamin B6	0.5	mg
Vitamin B12	1	μg
Vitamin C	30	mg
Folate	60	μg
Niacin	2	mg NE
Biotin	8	μg
Pantothenic Acid	1.4	mg
Calcium	160	mg
Phosphorous	140	mg
Iron	2.4	mg
Zinc	2.2	mg
Magnesium	60	mg
Potassium	320	mg
Iodine	30	μg
Manganese	0.7	mg
Copper	0.2	mg
Selenium	10	μg
Chromium	12	μg
Molybdenum	8	μg
Docosahexaenoic Acid + Eicosatetraenoic Acid	135	mg

Table S2. Detailed information on the included patients.

ID	Age	Sex	ECOG PS	height	Body weight before diagnosis	Body weight at the time of inclusion	Body weight change (%)	BMI at the time of inclusion	Diagnosis	Treatment setting	Chemotherapeutic agents	Group	Adherence rate
CNU-001	69	Male	0	162	55	48	12.73	18.27	CCC	Adjuvant	Capecitabine	Severe	100%
CNU-002	42	Male	1	174	84	67	20.24	22.21	CCC	Adjuvant	Concurrent chemo-radiotherapy with 5-Fluorouracil	Severe	100%
CNU-003	62	Female	0	161	63	50.4	20.00	19.44	Gallbladder cancer	Palliative	Gemcitabine/Cisplatin/ Nab-paclitaxel	Severe	100%
CNU-005	68	Female	0	150	58	51.5	21.67	22.86	Rectal cancer	Adjuvant	Capecitabine/Oxaliplatin	Severe	96.5%
CNU-006	57	Male	0	167	68	57	11.21	20.44	Colon cancer	Palliative	FOLFOX/Cetuximab	N-Severe	100%
CNU-007	74	Male	1	161	54	48	16.18	18.52	CCC	Palliative	Gemcitabine/Cisplatin	Severe	96%
CNU-008	69	Male	0	163	66	56	11.11	21.18	Colon cancer	Palliative	FOLFOX	N-Severe	75%
CNU-009	54	Female	0	164	96	87	15.15	32.91	Rectal cancer	Palliative	FOLFIRI/Bevacizumab	N-Severe	100%
CNU-011	58	Male	0	163	74	65	9.38	24.7	Gallbladder cancer	Palliative	Gemcitabine/Cisplatin	N-Severe	96.5%
CNU-012	76	Male	0	163	61	57	13.95	21.4	CCC	Adjuvant	Capecitabine	N-Severe	100%

ECOG PS, Eastern Cooperative Oncology Group Performance Status; BMI, body mass index; CCC, cholangiocarcinoma; FOLFOX, 5-Fluorouracil/oxaliplatin/leucovorin; FOLFIRI, 5-Fluorouracil/irinotecan/leucovorin

Table S3. Body composition analysis via DXA scan at baseline and 8 weeks according to cachexia severity.

Group	Characteristic	0 weeks	8 weeks	p-value*
Severe group	Body weight (kg)	52.40 (7.83)	52.04 (6.31)	0.82
	Lean body mass (kg)	35.51 (6.58)	36.14 (6.84)	0.16
	Bone mineral contents (kg)	1.96 (0.37)	1.93 (0.37)	0.22
	Fat mass (kg)	13.72 (3.45)	13.15 (2.84)	0.53
	Fat-free mass (kg)	37.47 (6.84)	38.07 (7.09)	0.19
	Fat-free mass index (kg/m^2)	14.26 (1.41)	14.48 (1.50)	0.19
	Lean body mass index (kg/m^2)	13.51 (1.31)	13.74 (1.41)	0.16
	Appendicular lean body mass index (kg/m^2)	5.19 (0.71)	5.19 (0.80)	0.96
Non-severe group	Body weight (kg)	64.20 (13.31)	65.48 (12.42)	0.18
	Lean body mass (kg)	40.74 (2.54)	40.97 (3.46)	0.75
	Bone mineral contents (kg)	1.94 (0.15)	1.93 (0.12)	0.82
	Fat mass (kg)	21.10 (13.13)	22.10 (12.12)	0.14
	Fat-free mass (kg)	42.68 (2.60)	42.90 (3.49)	0.76
	Fat-free mass index (kg/m^2)	15.89 (1.23)	15.97 (1.55)	0.75
	Lean body mass index (kg/m^2)	15.17 (1.19)	15.25 (1.53)	0.74
	Appendicular lean body mass index (kg/m^2)	5.82 (0.72)	6.12 (0.89)	0.08

* Wilcoxon signed-rank test

Table S4. Changes in biochemical markers at baseline, after 4 weeks, and 8 weeks according to cachexia severity.

Group	Characteristic	Normal range	Severe N = 5*	Non-severe N = 5*	p-value†
Baseline (Week 0)	WBC (10^3/µL)	4.2–9.5	10.26 (2.64)	9.27 (4.65)	0.7
	Lymphocyte (10^3/µL)	1.0–4.8	2.09 (0.42)	1.70 (0.74)	0.4
	Neutrophil (10^3/µL)	1.5–8.0	4.57 (2.15)	6.46 (3.76)	0.3
	Hemoglobin (g/dL)	12.3–16.7	12.80 (3.90)	11.40 (2.41)	0.8
	Platelet (10^3/µL)	154–384	383.20 (96.79)	291.00 (113.55)	0.2
	Total Protein (g/dL)	6.5–8.0	7.38 (0.50)	6.56 (0.64)	0.059
	Albumin (g/dL)	4.0–5.0	3.80 (0.84)	3.60 (0.55)	0.8
	Vitamin D (ng/mL)	30–100	14.60 (7.09)	13.60 (8.29)	0.5
	CRP (mg/dL)	0–0.5	5.18 (5.21)	2.34 (3.39)	0.7
	Interleukin-6 (pg/mL)	0–7	23.00 (25.14)	25.40 (29.84)	0.8
	GDF-15 (pg/mL)	200–1200	978.05 (556.52)	919.68 (742.49)	0.7
	Neutrophil-to-lymphocyte ratio		2.10 (0.79)	4.31 (3.05)	0.2
	Platelet-to-lymphocyte ratio		192.54 (80.67)	188.80 (80.62)	0.8
Week 4	WBC (10^3/µL)	4.2–9.5	8.09 (2.32)	4.98 (2.92)	0.10
	Lymphocyte (10^3/µL)	1.0–4.8	1.49 (0.91)	1.49 (0.62)	>0.9
	Neutrophil (10^3/µL)	1.5–8.0	4.87 (2.66)	2.60 (2.35)	0.2
	Hemoglobin (g/dL)	12.3–16.7	11.20 (2.68)	11.20 (1.64)	>0.9
	Platelet (10^3/µL)	154–384	348.00 (131.34)	246.60 (136.28)	0.4
	Total Protein (g/dL)	6.5–8.0	6.82 (0.71)	6.58 (0.55)	0.7
	Albumin (g/dL)	4.0–5.0	3.60 (0.89)	3.60 (0.55)	>0.9
	Vitamin D (ng/mL)	30–100	15.60 (10.21)	16.80 (7.79)	0.7

	CRP (mg/dL)	0–0.5	7.56 (9.13)	1.08 (1.00)	0.3
	Interleukin-6 (pg/mL)	0–7	31.80 (26.38)	108.00 (196.93)	>0.9
	GDF-15	200–1200	982.26 (665.63)	814.97 (634.81)	>0.9
	Neutrophil-to-lymphocyte ratio		4.16 (2.69)	1.76 (1.69)	0.2
	Platelet-to-lymphocyte ratio		275.74 (111.71)	164.50 (43.89)	0.2
Week 8	WBC (10^3/μL)	4.2–9.5	7.58 (3.58)	5.12 (1.01)	0.4
	Lymphocyte (10^3/μL)	1.0–4.8	2.53 (1.85)	1.48 (0.60)	0.5
	Neutrophil (10^3/μL)	1.5–8.0	2.85 (1.15)	2.79 (0.89)	>0.9
	Hemoglobin (g/dL)	12.3–16.7	11.40 (2.51)	11.80 (1.30)	>0.9
	Platelet (10^3/μL)	154–384	256.60 (82.45)	190.20 (74.81)	0.2
	Total Protein (g/dL)	6.5–8.0	6.64 (1.23)	6.64 (0.54)	>0.9
	Albumin (g/dL)	4.0–5.0	3.60 (0.55)	4.00 (0.00)	0.2
	Vitamin D (ng/mL)	30–100	12.80 (3.11)	17.20 (9.42)	0.7
	CRP (mg/dL)	0–0.5	1.84 (3.40)	0.38 (0.29)	0.7
	Interleukin-6 (pg/mL)	0–7	14.00 (12.21)	8.20 (6.14)	0.6
	GDF-15	200–1200	1,031.71 (505.20)	667.95 (312.71)	0.4
	Neutrophil-to-lymphocyte ratio		1.80 (1.44)	2.20 (1.33)	0.4
	Platelet-to-lymphocyte ratio		152.75 (95.95)	131.28 (30.99)	0.8

*Mean (SD)

†Mann-Whitney U test

Table S5. Changes in quality of life and symptom scores (EORTC QLQ-C30) across treatment periods by cachexia severity group.

	Baseline (Week 0)			Week 4			Week 8		
Characteristic	Severe N = 5*	Non-severe N = 5*	p-value†	Severe N = 5*	Non-severe N = 5*	p-value†	Severe N = 5*	Non-severe N = 5*	p-value†
Physical function	82.67 (13.82)	90.67 (10.11)	0.4	81.33 (14.45)	89.33 (8.94)	0.4	85.33 (8.69)	89.33 (8.94)	0.5
Role function	86.67 (18.26)	93.33 (14.91)	0.6	80.00 (18.26)	93.33 (14.91)	0.3	86.67 (18.26)	90.00 (14.91)	>0.9
Emotional	90.00 (13.69)	88.33 (16.24)	>0.9	83.33 (10.21)	93.33 (10.87)	0.2	88.33 (11.18)	93.33 (10.87)	0.6
Cognitive	86.67 (13.94)	90.00 (14.91)	0.7	80.00 (13.94)	86.67 (13.94)	0.5	83.33 (16.67)	90.00 (14.91)	0.6
Social	73.33 (14.91)	80.00 (18.26)	0.6	73.33 (14.91)	80.00 (18.26)	0.6	73.33 (14.91)	80.00 (18.26)	0.6
Symptom scales									
Fatigue	28.89 (9.94)	15.56 (16.85)	0.2	35.56 (9.30)	20.00 (12.17)	0.080	28.89 (12.67)	24.44 (9.30)	0.6
Nausea/Vomiting	0.00 (0.00)	3.33 (7.45)	0.4	10.00 (14.91)	6.67 (9.13)	>0.9	6.67 (14.91)	6.67 (9.13)	0.8
Pain	13.33 (13.94)	6.67 (9.13)	0.5	20.00 (18.26)	13.33 (29.81)	0.5	16.67 (11.79)	10.00 (14.91)	0.4

Dyspnea	6.67 (14.91)	6.67 (14.91)	>0.9	6.67 (14.91)	0.00 (0.00)	0.4	0.00 (0.00)	0.00 (0.00)	
Insomnia	26.67 (27.89)	13.33 (18.26)	0.5	26.67 (27.89)	20.00 (18.26)	0.8	20.00 (18.26)	13.33 (18.26)	0.6
Anorexia	40.00 (14.91)	26.67 (14.91)	0.2	26.67 (14.91)	20.00 (18.26)	0.6	26.67 (14.91)	20.00 (18.26)	0.6
Constipation	33.33 (33.33)	20.00 (29.81)	0.6	20.00 (29.81)	20.00 (29.81)	>0.9	13.33 (29.81)	20.00 (29.81)	0.7
Diarrhea	6.67 (14.91)	0.00 (0.00)	0.4	13.33 (29.81)	0.00 (0.00)	0.4	20.00 (29.81)	0.00 (0.00)	0.2
Financial difficulties	26.67 (14.91)	20.00 (18.26)	0.6	26.67 (14.91)	20.00 (18.26)	0.6	26.67 (14.91)	20.00 (18.26)	0.6
Global health status / QoL	53.33 (7.45)	50.00 (16.67)	0.8	50.00 (0.00)	53.33 (13.94)	0.6	50.00 (11.79)	56.67 (9.13)	0.4

*Mean (SD), †Mann-Whitney U test

Table S6. Variable Importance in Projection (VIP) scores of metabolites associated with differential metabolic response to ONS intervention in cancer cachexia (VIP > 1.0).

Metabolite	Matched name	HMDB	PubChem	KEGG	VIP Component 1	VIP Component 2
**C20:2**	Eicosadienoic acid	HMDB0005060	6439848	C16525	2.957979	2.452913
**C20:1**	9Z-Eicosenoic acid	HMDB0062436	5282767	NA	2.919742	2.418999
**C18:1**	Oleic acid	HMDB0000207	445639	C00712	2.911627	2.432013
**C18:2**	Linoleic acid	HMDB0000673	5280450	C01595	2.907371	2.431395
**C16:1**	Palmitoleic acid	HMDB0003229	445638	C08362	2.816997	2.363833
**C16:0**	Palmitic acid	HMDB0000220	985	C00249	2.729573	2.296943
**C16:2**	7Z,10Z-Hexadecadienoic acid	HMDB0000477	13932172	NA	2.700545	2.251238
**C18:0**	Stearic acid	HMDB0000827	5281	C01530	2.52905	2.11914
**1-3-Dimethyluracil**	1,3-Dimethyluracil	HMDB0002144	70122	NA	2.488343	2.027201
**Cholic acid**	Cholic acid	HMDB0000619	221493	C00695	2.063579	1.746063

**3-Cresotinic acid**	3-Cresotinic acid	HMDB0002390	6738	C14088	1.971914	1.604379
**Hyodeoxycholic acid**	Hyodeoxycholic acid	HMDB0000733	9963687	C15517	1.884425	1.590132
**2-Phenylpropionic acid**	2-Phenylpropionate	HMDB0011743	10296	NA	1.787021	1.544915
**C20:0**	Arachidic acid	HMDB0002212	10467	C06425	1.64459	1.362371
**3-methylphenylacetic acid**	3-Methylphenylacetic acid	HMDB0002222	12121	NA	1.529506	1.289933
**Myristic acid**	Myristic acid	HMDB0000806	11005	C06424	1.388374	1.135439
**Stearic acid**	Stearic acid	HMDB0000827	5281	C01530	1.366365	1.457056
**2-Phenylbutyric acid**	2-Phenylbutyric acid	HMDB0000329	7012	NA	1.339105	1.11114
**Sebacic acid**	Sebacic acid	HMDB0000792	5192	C08277	1.322484	1.136929
**dihydroorotate**	4,5-Dihydroorotic acid	HMDB0000528	648	C00337	1.316283	1.11936
**Hexanoyl Glycine**	Hexanoylglycine	HMDB0000701	99463	NA	1.206788	1.01653

**1-Methyl-hydantoin**	N-Methylhydantoin	HMDB0003646	69217	C02565	1.19692	1.028833
**Gentisic acid**	Gentisic acid	HMDB0000152	3469	C00628	1.184815	0.973419
**fumarate**	Fumaric acid	HMDB0000134	444972	C00122	1.151774	1.059618
**N-Propionylglycine**	Propionylglycine	HMDB0000783	98681	NA	1.139437	1.020255
**creatine**	Creatine	HMDB0000064	586	C00300	1.083013	1.188116
**Linoleic acid**	Linoleic acid	HMDB0000673	5280450	C01595	1.075781	0.944093
**N-Acetylglycine**	Acetylglycine	HMDB0000532	10972	NA	1.024787	0.94881
**N-Butyrylglycine**	N-Butyrylglycine	HMDB0000808	88412	NA	1.008624	0.90222
**malate**	Malic acid	HMDB0000156	222656	C00149	1.007557	0.974614

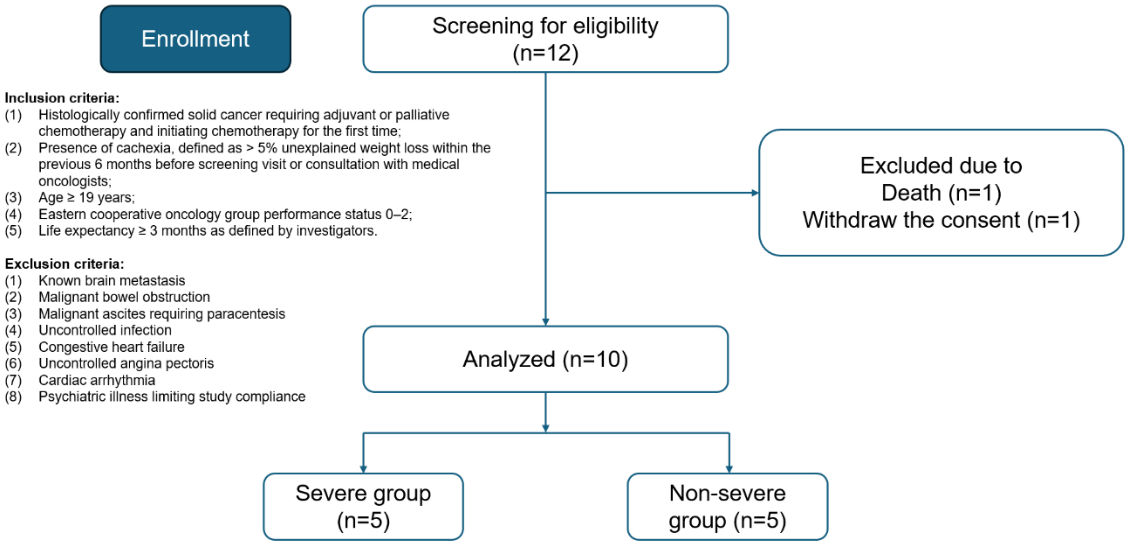

Figure S1. CONSORT diagram of this study.

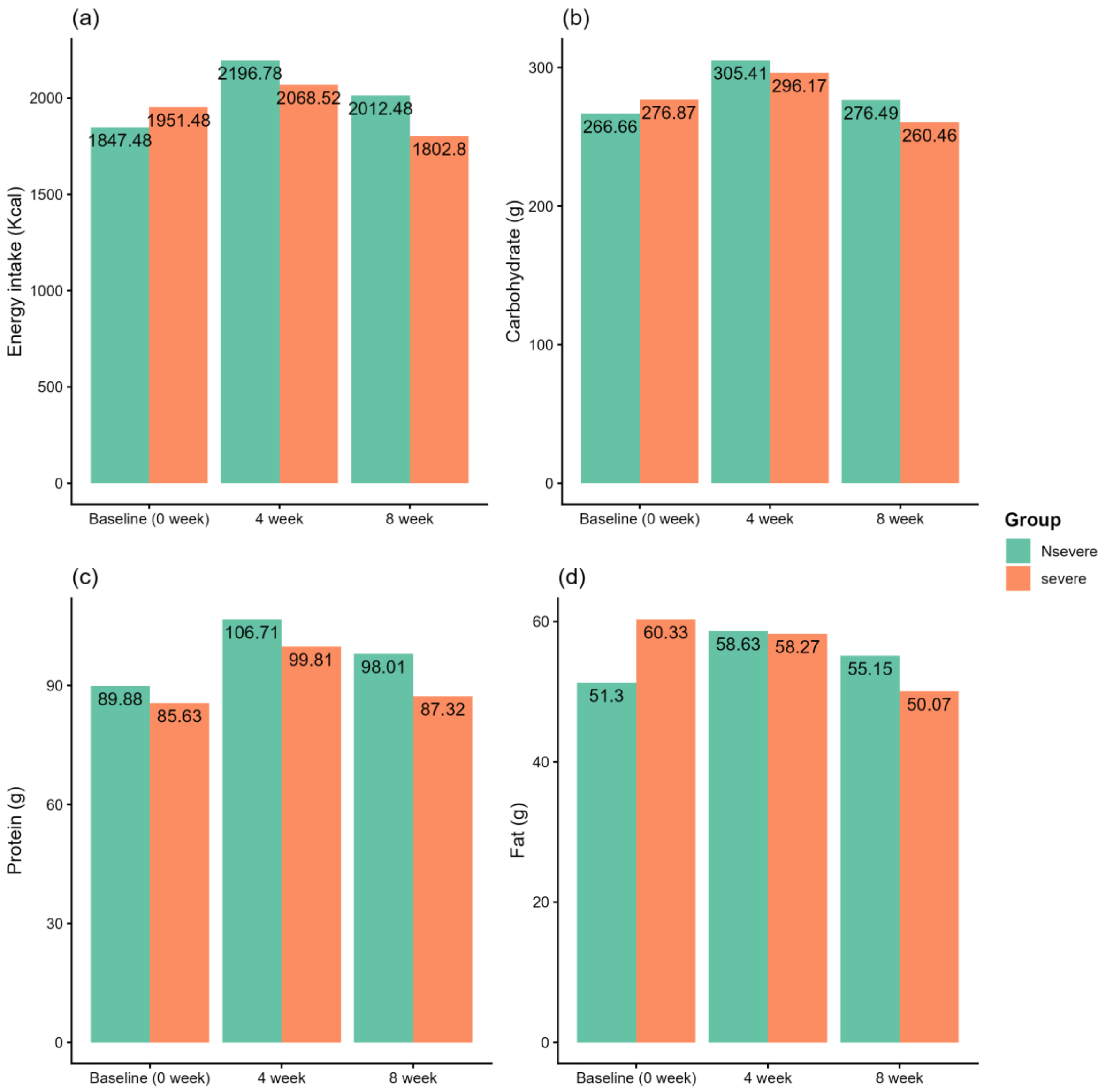

Figure S2. Changes in dietary intakes by cachexia severity group. (a) Total energy intake (b) carbohydrate (c) protein, and (d) fat.

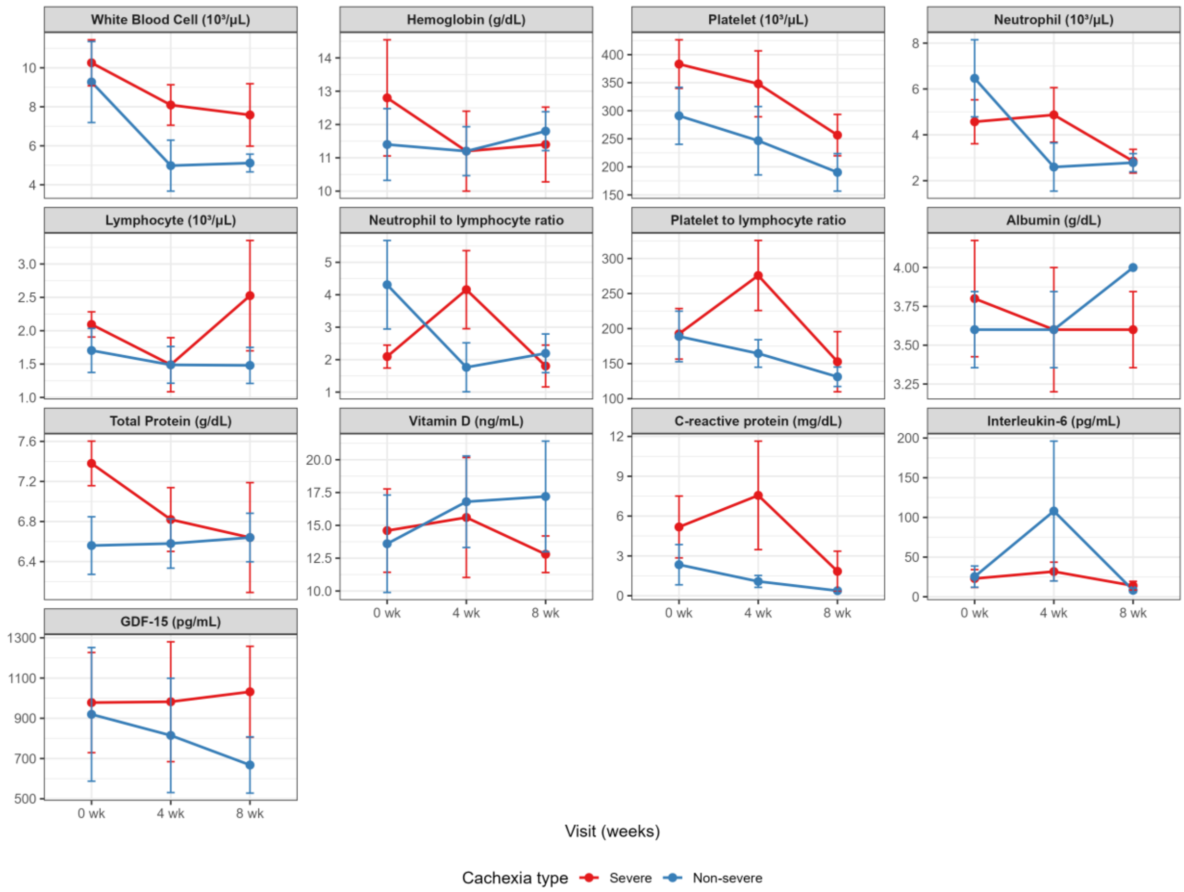

Figure S3. Changes in biochemical markers by cachexia severity group.

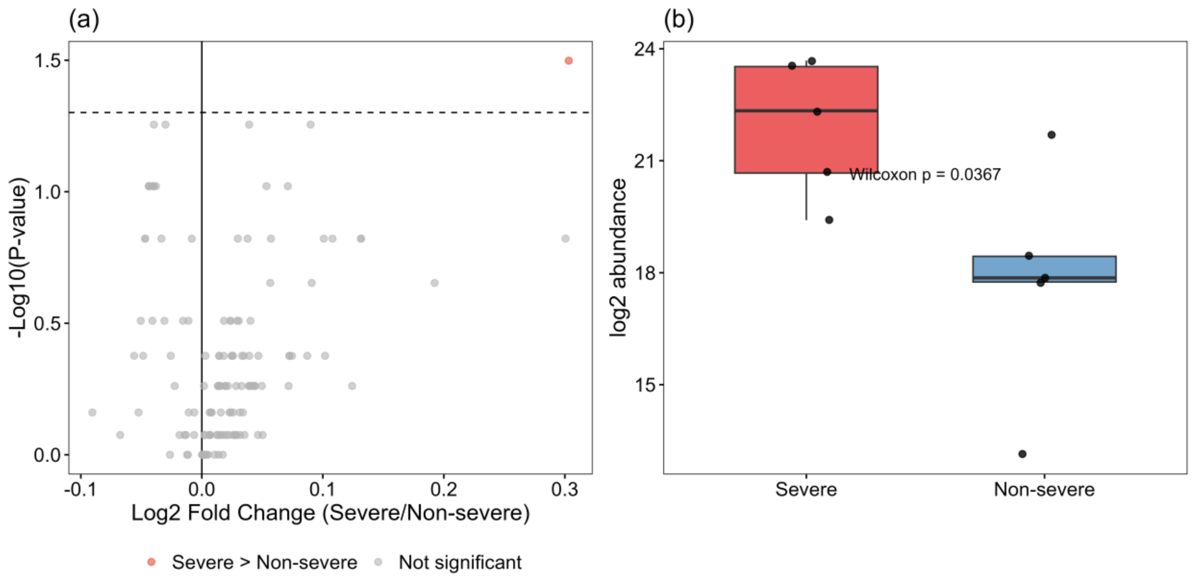

Figure S4. Results of serum metabolite analysis. (**a**) Volcano plots at baseline. (**b**) Levels of hyodeoxycholic acid according to cachexia severity group.

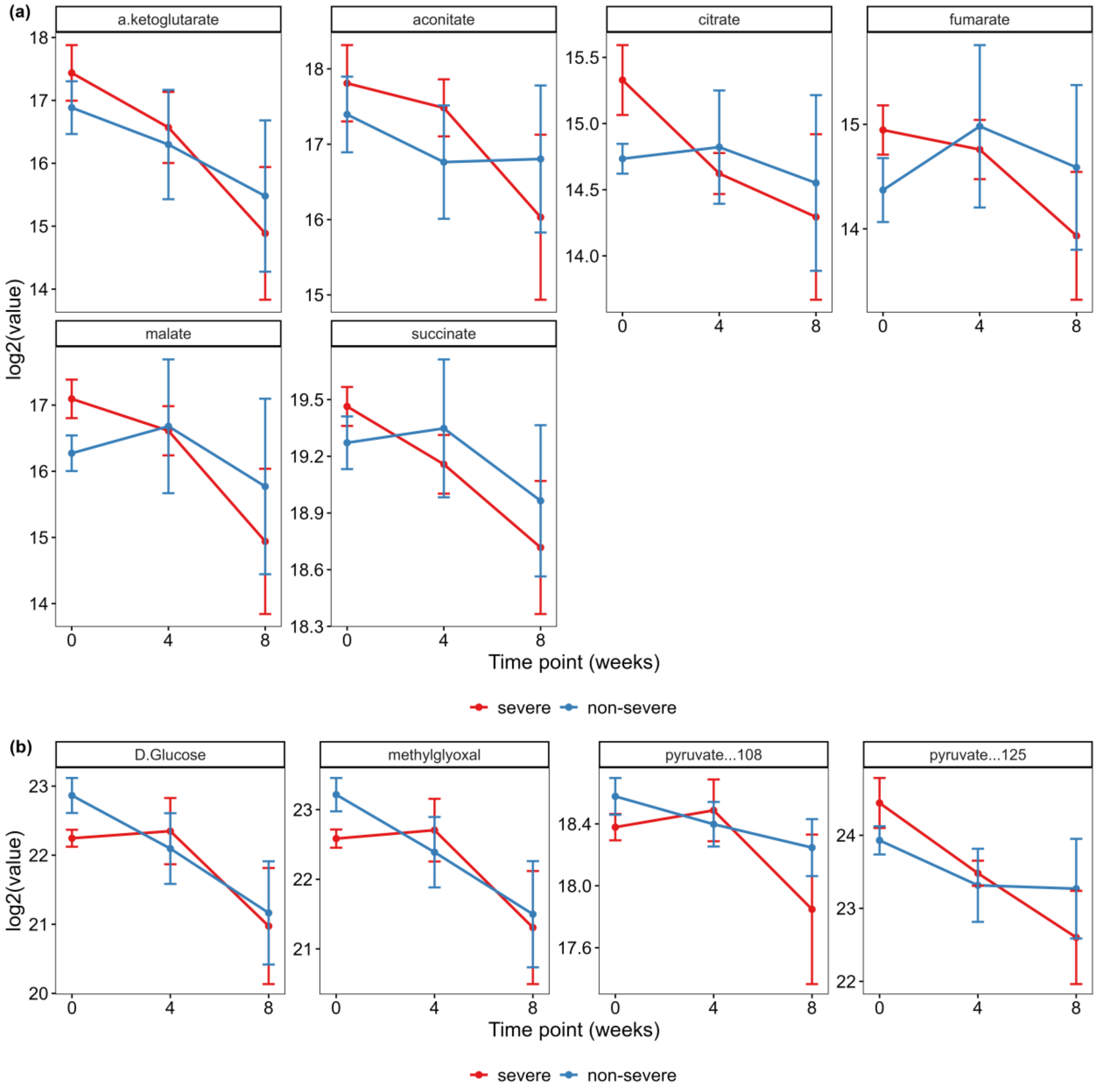

Figure S5. Results of serum metabolite analysis: (**a**) Changes in tricarboxylic acid cycle intermediates by Cachexia Severity Group; (**b**) Glucose metabolism intermediates by Cachexia Severity Group.

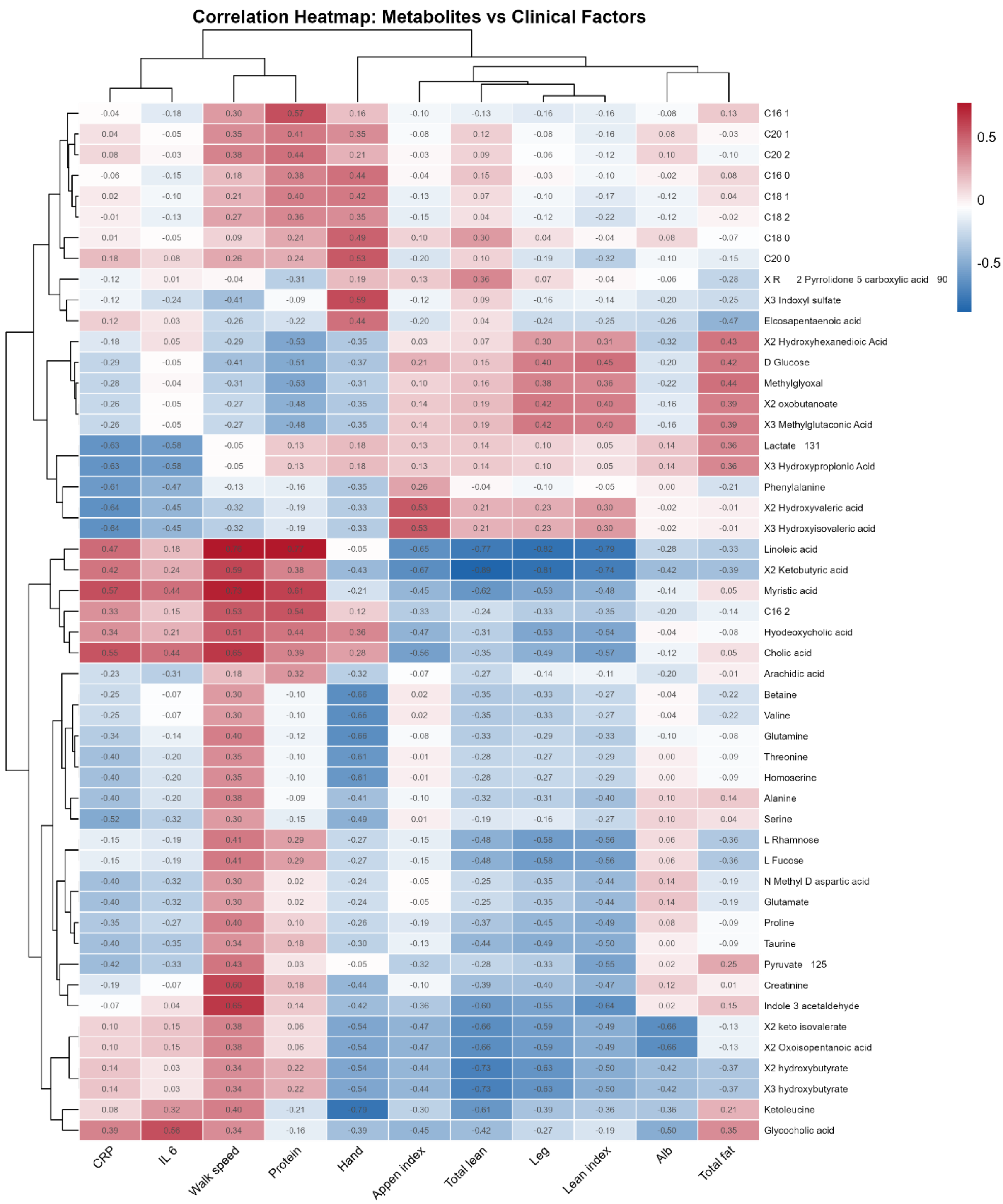

Figure S6. Correlation heatmap between serum metabolites and clinical parameters. The heatmap illustrates Spearman's rank correlation coefficients between the top 50 serum metabolites (ranked by variance) and key clinical factors related to cancer cachexia. Red and blue colors represent positive and negative correlations, respectively, as indicated by the color scale bar. Numbers within the cells indicate the correlation coefficient (*r*). Clinical parameters include calf circumference (leg), handgrip strength (hand), walking speed, and biochemical markers (albumin, CRP, and IL-6).

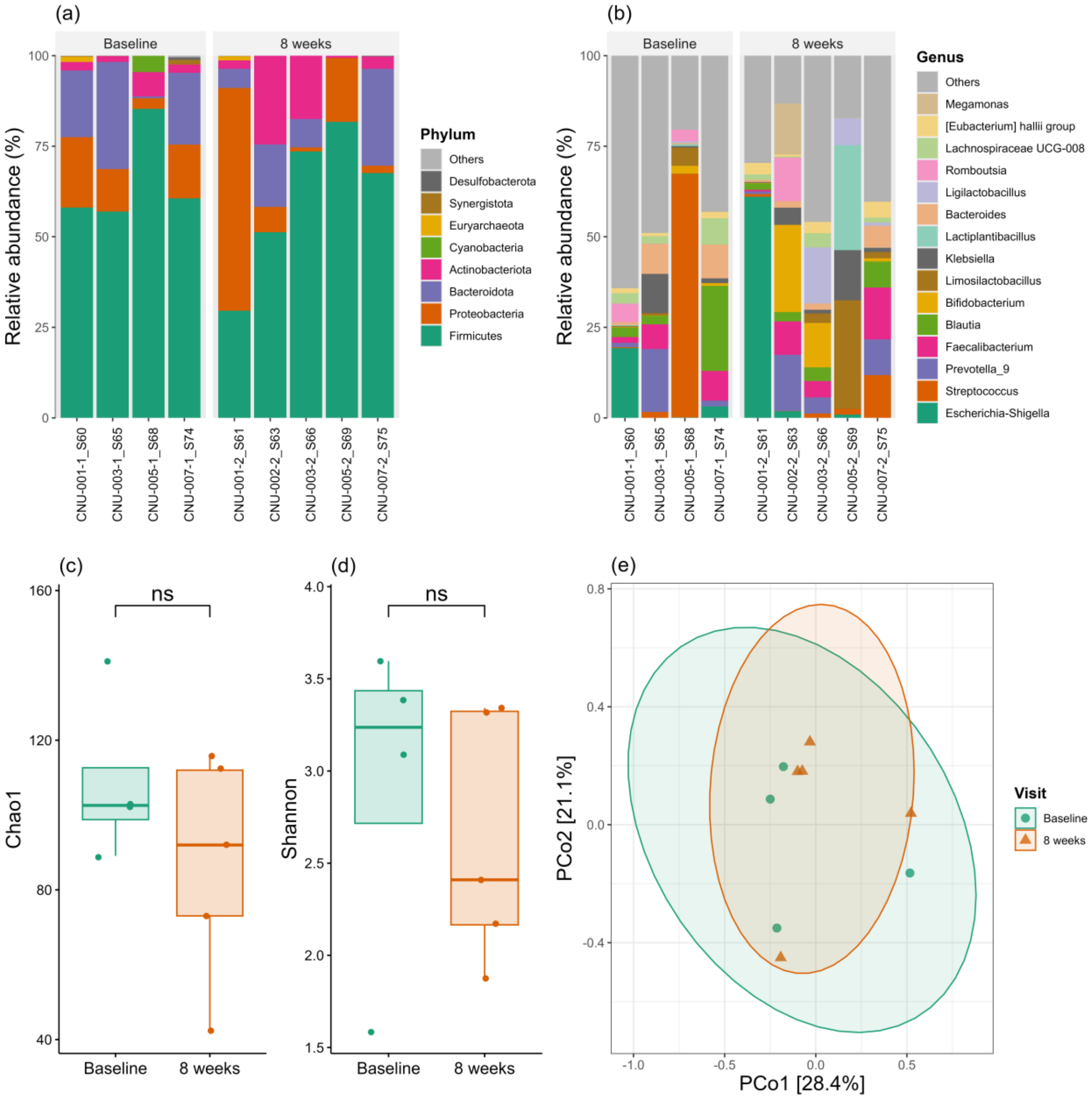

Figure S7. Gut Microbiome profiles and α/β diversity analysis between baseline and after 8 weeks of ONS administration in the severe group: (**a**) Phylum-level taxonomic composition showing the relative abundance of the eight most abundant bacterial phyla at baseline and after 8 weeks; (**b**) Genus-level taxonomic composition displaying the relative abundance of the fifteen most abundant bacterial genera; (**c**) Chao1 richness index; (**d**) Shannon diversity index; (**e**) Principal coordinate analysis (PCoA) plot based on Bray-Curtis dissimilarity.

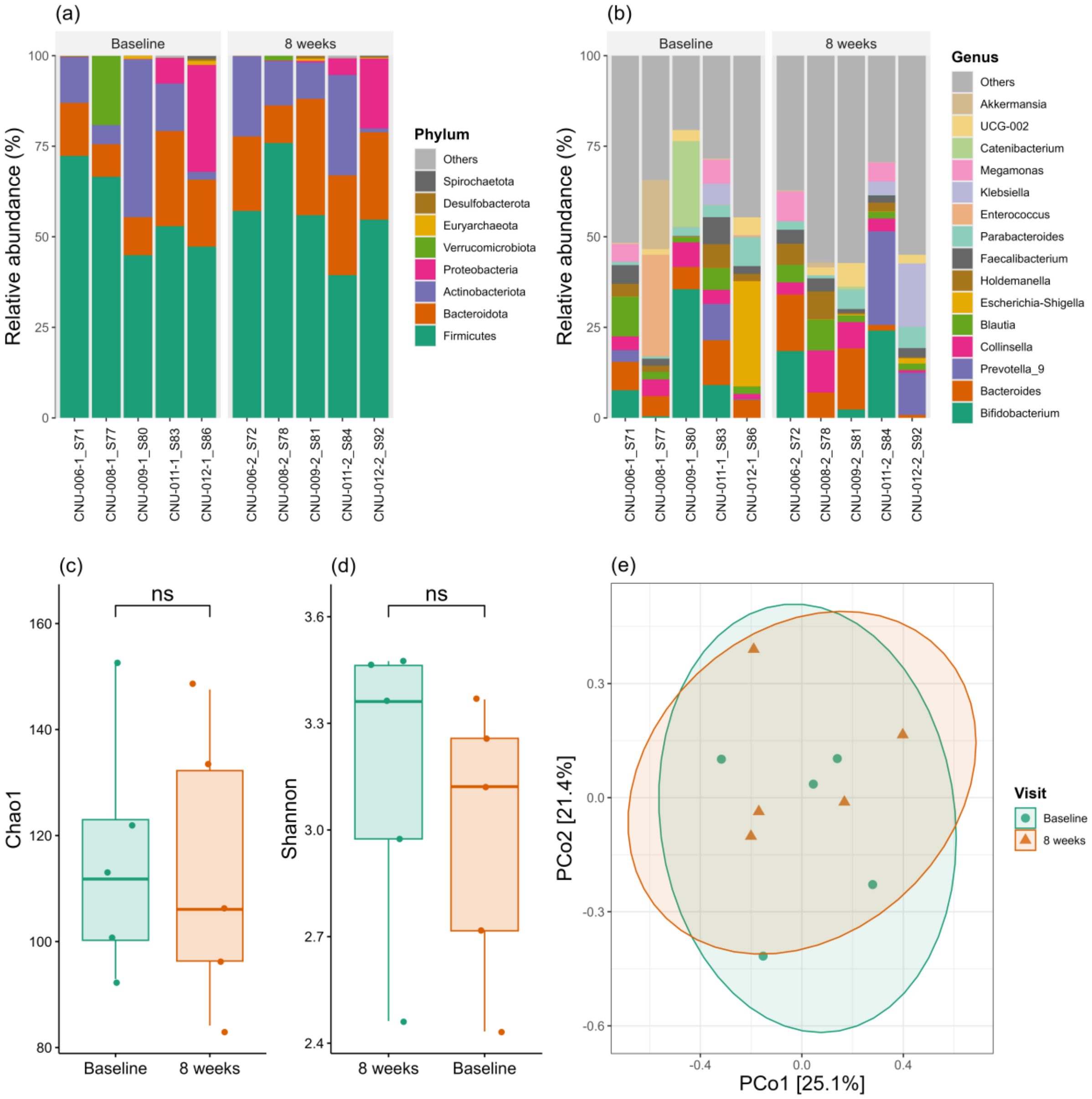

Figure S8. Gut microbiome profiles and α/β diversity analysis between baseline and after 8 weeks of ONS administration in the non-severe group: (**a**) Phylum-level taxonomic composition showing the relative abundance of the eight most abundant bacterial phyla at baseline and 8 weeks; (**b**) Genus-level taxonomic composition displaying the relative abundance of the fifteen most abundant bacterial genera; (**c**) Chao1 richness index; (**d**) Shannon diversity index; (**e**) Principal coordinate analysis (PCoA) plot based on Bray-Curtis dissimilarity.
